# Cardiovascular outcomes with sodium–glucose cotransporter-2 inhibitors vs other glucose-lowering drugs in 13 countries across three continents: analysis of CVD-REAL data

**DOI:** 10.1186/s12933-021-01345-z

**Published:** 2021-07-31

**Authors:** Kamlesh Khunti, Mikhail Kosiborod, Dae Jung Kim, Shun Kohsaka, Carolyn S. P. Lam, Su-Yen Goh, Chern-En Chiang, Jonathan E. Shaw, Matthew A. Cavender, Navdeep Tangri, Josep Franch-Nadal, Reinhard W. Holl, Marit E. Jørgensen, Anna Norhammar, Johan G. Eriksson, Francesco Zaccardi, Avraham Karasik, Dianna J. Magliano, Marcus Thuresson, Hungta Chen, Eric Wittbrodt, Johan Bodegård, Filip Surmont, Peter Fenici, the CVD-REAL Investigators and Study Group, Mikhail Kosiborod, Matthew A. Cavender, John P. Wilding, Kamlesh Khunti, Anna Norhammar, Kåre Birkeland, Marit Eika Jørgensen, Reinhard W. Holl, Carolyn S. P. Lam, Hanne Løvdal Gulseth, Bendix Carstensen, Esther Bollow, Josep Franch-Nadal, Luis Alberto García Rodríguez, Avraham Karasik, Navdeep Tangri, Shun Kohsaka, Dae Jung Kim, Jonathan Shaw, Suzanne Arnold, Su-Yen Goh, Chern-En Chiang, Johan G. Eriksson, Francesco Zaccardi, Peter Fenici, Johan Bodegård, Hungta Chen, Filip Surmont, Rachel Kendrick, Wesley Belli, Eric T. Wittbrodt, Matthias Saathoff, Yusuke Noguchi, Donna Tan, Maro Williams, Hye Won Lee, Maya Greenbloom, Oksana Kaidanovich-Beilin, Karolina Andersson-Sundell, Khung Keong Yeo, Yong Mong Bee, Joan Khoo, Agnes Koong, Yee How Lau, Fei Gao, Wee Boon Tan, Hanis Abdul Kadir, Kyoung Hwa Ha, Jinhee Lee, Gabriel Chodick, Cheli Melzer Cohen, Reid Whitlock, Lucia Cea Soriano, Oscar Fernándex Cantero, Jordan A. Menzin, Matthew Guthrie, Jennie Ilomaki, Dianna Magliano, Fabian Hoti, Solomon Christopher, Minna Vehkala

**Affiliations:** 1 University of Leicester, University Road, Leicester, LE1 7RH UK; 2 Saint Luke’s Mid America Heart Institute, Kansas City, MO 64111 USA; 3 University of Missouri-Kansas City, Kansas City, MO 64110 USA; 4 The George Institute for Global Health, Sydney, NSW 2042 Australia; 5 Ajou University School of Medicine, Suwon, 16499 Republic of Korea; 6 Keio University School of Medicine, Tokyo, 160-8582 Japan; 7 National Heart Center Singapore, Singapore, 169609 Singapore; 8 SingHealth Duke-NUS, Singapore, 168753 Singapore; 9 University Medical Center Groningen, Groningen, The Netherlands; 10 Singapore General Hospital, Singapore, 169608 Singapore; 11 General Clinical Research Center, Division of Cardiology, Taipei Veterans General Hospital, Taipei, Taiwan; 12 National Yang-Ming University, Taipei, Taiwan; 13 Baker Heart and Diabetes Institute, Melbourne, VIC 3004 Australia; 14 University of North Carolina, Chapel Hill, NC 27599 USA; 15 University of Manitoba, Winnipeg, MB Canada; 16 Institut Universitari D’investigació en Atenció Primaria (IDIAP Jordi Gol), CIBERDEM, Barcelona, Spain; 17 Institute of Epidemiology and Medical Biometry, ZIBMT, University of Ulm, 89081 Ulm, Germany; 18 Steno Diabetes Center Copenhagen, 2820 Gentofte, Denmark; 19 University of Southern Denmark, Copenhagen, Denmark; 20 Karolinska Institutet, 171 76 Stockholm, Sweden; 21 Department of General Practice and Primary Health Care, University of Helsinki and Helsinki University Hospital, 00290 Helsinki, Finland; 22 Folkhälsan Research Center, Helsinki, Finland; 23 Department of Obstetrics & Gynecology, Yong Loo Lin School of Medicine, National University of Singapore, Singapore Institute for Clinical Sciences (SICS), Agency for Science, Technology and Research (A*STAR), Singapore, Singapore; 24 Tel Aviv University, Tel Aviv, Israel; 25 School of Public Health and Preventive Medicine, Monash University, Baker Heart and Diabetes Institute, Melbourne, VIC 3004 Australia; 26 Statisticon AB, Uppsala, Sweden; 27 BioPharmaceuticals R&D, AstraZeneca, Gaithersburg, MD 20878 USA; 28 BioPharmaceuticals Medical, AstraZeneca, Gaithersburg, MD 20878 USA; 29 BioPharmaceuticals R&D, AstraZeneca, 0663 Oslo, Norway; 30 BioPharmaceuticals Medical, AstraZeneca, Cambridge, CB2 8PA UK; 31 Cardiovascular, Renal and Metabolism, BioPharmaceuticals Medical, AstraZeneca, Cambridge, CB2 8PA UK

**Keywords:** Sodium–glucose cotransporter-2 inhibitors, Cardiovascular outcomes, Heart failure, Type 2 diabetes

## Abstract

**Background:**

Randomized, controlled cardiovascular outcome trials may not be fully representative of the management of patients with type 2 diabetes across different geographic regions. We conducted analyses of data from the multinational CVD-REAL consortium to determine the association between initiation of sodium–glucose cotransporter-2 inhibitors (SGLT-2i) and cardiovascular outcomes, including subgroup analyses based on patient characteristics.

**Methods:**

De-identified health records from 13 countries across three continents were used to identify patients newly-initiated on SGLT-2i or other glucose-lowering drugs (oGLDs). Propensity scores for SGLT-2i initiation were developed in each country, with 1:1 matching for oGLD initiation. In the matched groups hazard ratios (HRs) for hospitalization for heart failure (HHF), all-cause death (ACD), the composite of HHF or ACD, myocardial infarction (MI) and stroke were estimated by country, and pooled using a weighted meta-analysis. Multiple subgroup analyses were conducted across patient demographic and clinical characteristics to examine any heterogeneity in treatment effects.

**Results:**

Following matching, 440,599 new users of SGLT-2i and oGLDs were included in each group. Mean follow-up time was 396 days for SGLT-2i initiation and 406 days for oGLDs initiation. SGLT-2i initiation was associated with a lower risk of HHF (HR: 0.66, 95%CI 0.58–0.75; p < 0.001), ACD (HR: 0.52, 95%CI 0.45–0.60; p < 0.001), the composite of HHF or ACD (HR: 0.60, 95%CI 0.53–0.68; p < 0.001), MI (HR: 0.85, 95%CI 0.78–0.92; p < 0.001), and stroke (HR: 0.78, 95%CI 0.72–0.85; p < 0.001); regardless of patient characteristics, including established cardiovascular disease, or geographic region.

**Conclusions:**

This CVD-REAL study extends the findings from the SGLT-2i clinical trials to the broader setting of an ethnically and geographically diverse population, and across multiple subgroups.

*Trial registration* NCT02993614

**Supplementary Information:**

The online version contains supplementary material available at 10.1186/s12933-021-01345-z.

## Background

Despite recent improvements, cardiovascular disease (CVD) remains the leading global cause of mortality and morbidity in patients with type 2 diabetes [1]. The prevalence of diabetes continues to increase, with the majority of people affected residing in the Asia–Pacific region, South East Asia and the Middle East [2–4]. However, global outcomes data are sparse for new therapies in people with type 2 diabetes, especially outside North America and Europe. Previous studies have shown large variations in the management of people with diabetes and the prevalence of complications [5, 6]. More recently, global collaborative studies have shown that there are differences in patient characteristics, treatment patterns, and types of adverse CVD events experienced by patients in different regions and different ethnicities [7]. Moreover, results of randomized controlled cardiovascular outcome trials have been criticised for not being representative of the management of people with type 2 diabetes in real-world settings [8], with most patients being recruited in North America and Europe. Clinicians across other world regions are therefore cautious about generalizing the results of trials into their clinical practice.

Over recent years, there has been a number of studies reporting the comparative effectiveness of novel therapies in the ‘real-world’ setting. Sodium–glucose cotranspoter-2 inhibitors (SGLT-2is) have now been studied in several cardiovascular outcome trials [9–13], with a recent systematic review and meta-analysis showing the benefits of this class of drugs on major cardiovascular events in people with established CVD, and on heart failure and renal outcomes in people with and without CVD [14]. We have previously conducted a number of pharmaco-epidemiological studies (CVD-REAL—Comparative Effectiveness of Cardiovascular Outcomes in New Users of Sodium–Glucose Cotransporter-2 Inhibitors) on the effectiveness of SGLT-2i in the real-world setting [15, 16]. Although there were differences in point estimates across countries for some outcomes, the directionality of associations was consistent despite variable patient characteristics, health care settings, practice patterns, and specific SGLT-2i compounds used.

In this study, we conducted further analyses of the largest CVD-REAL dataset to date, to assess a broad range of cardiovascular outcomes, including hospitalization for heart failure, all-cause mortality, myocardial infarction, and stroke. Our additional aim was to use the large number of patients and events in this dataset to expand the results of CVD-REAL by examining SGLT2i effects across a broad range of subgroups including baseline therapies and other chronic multimorbidities. We also report on the time trends of SGLT-2i prescribing in the 13 included countries.

## Methods

### Data sources

Analyses were conducted on de-identified health records from 13 countries (Australia, Canada, Denmark, Finland, Germany, Israel, Japan, Singapore, South Korea, Spain, Sweden, Taiwan, and the USA). Descriptions of the sources of data have been reported previously [16–19], apart from Finland which is included in Additional file: Data Sources 1.

### Patient cohort

Patients with type 2 diabetes were identified using standard diagnosis codes (Additional file 1: Table S1), except in Australia where this was based on physician or nurse educator clinical diagnosis of type 2 diabetes. All episodes of new initiation of either SGLT-2i or other glucose-lowering drugs (oGLD) were selected within the country-specific date range for availability of SGLT-2i [Additional file 1: Table S2; range: December 1, 2012 (Denmark) to May 1, 2016 (Taiwan)]. Treatment initiation episodes were defined as written/dispensed prescription (as initial or add-on therapy) for any SGLT-2i or oGLD, including fixed-dose combinations, without any use of the same therapy during the preceding 12 months; a patient might contribute with several episodes, every time the criteria of new user is met. Additional inclusion criteria were: age ≥ 18 years on the index date (defined as the prescription date for new initiation of an SGLT-2i or oGLD) and availability of historic data for more than 1 year in the database before the index date. Patients with type 1 or gestational diabetes were excluded. Patients were followed from the index date until migration/leaving the practice/database, last date of data collection, outcome date, or censoring date [Additional file 1: Table S2; range: December 31, 2014 (Australia) to November 30, 2017 (Singapore)].

### Outcomes

The outcomes were hospitalization for heart failure (HHF), all-cause death, the composite of HHF or all-cause death, non-fatal myocardial infarction, or non-fatal stroke. Outcomes were defined using primary discharge diagnosis codes (Additional file 1: Table S3) and validated independently in each country. Data regarding all-cause death were available from all countries; data for the other outcomes were available from all countries except Australia. Additionally, in Japan and Singapore only information on in-hospital deaths was available; however, in-hospital deaths represent the majority of fatal events in these countries according to the national statistics [20, 21]. In Sweden and Denmark, HHF was defined by any hospital visit with a registered main diagnosis of heart failure [inpatient or outpatient visit; defined using diagnosis codes (Additional file 1: Table S3)].

### Statistical analysis

Detailed statistical methods are described in a prior publication [15]. Briefly, baseline characteristics were analyzed using descriptive statistics. Categorical variables were described by frequencies and percentages, and mean ± standard deviation (SD) were used for continuous variables. The overall mean across all databases was a summary estimate of country-specific means, weighted according to the number of patients in each database. The proportion of exposure time contributed by individual agents was summarized both overall and by country.

A non-parsimonious propensity score for initiating SGLT-2i was developed separately for each country. All available variables in each country that could affect treatment assignment or outcomes were included in the propensity score (Additional file 1: Table S4; baseline comorbidity information was not available for Australia, although extensive medication data were available). Based on propensity scores, episodes of patients initiating SGLT-2i were matched 1:1 with episodes of initiating oGLD. The adequacy of matching was assessed by evaluating post-match standardized differences in patient characteristics (Table 1). A non-negligible imbalance was considered if a > 10% standardized difference occurred between the two groups post-match.

**Table 1:** Baseline characteristics for all 13 countries combined (post-match)

	SGLT-2 inhibitor (N = 440,599)	oGLD (N = 440,599)	std diff (%)
Age, years	58.0 (11.6)	57.8 (12.7)	1.4
Women	193,825 (44.0)	194,123 (44.1)	0.1
CV-history	134,331 (31.5)	129,993 (30.5)	2.2
Myocardial infarction	19,346 (4.5)	18,968 (4.4)	0.4
Unstable angina	22,030 (5.2)	21,489 (5.0)	0.6
Heart failure	32,736 (7.7)	31,948 (7.5)	0.7
Atrial fibrillation	18,448 (4.3)	17,910 (4.2)	0.6
Stroke	47,437 (11.1)	45,353 (10.6)	1.6
PAD	22,391 (5.2)	22,054 (5.2)	0.4
Microvascular disease	218,288 (51.1)	214,311 (50.2)	1.9
CKD	24,528 (5.7)	23,969 (5.6)	0.6
Frailty (yes)*	36,736 (8.9)	36,498 (8.8)	0.2
Glucose-lowering therapies			
Metformin	334,441 (75.9)	335,731 (76.2)	0.7
SU	197,712 (44.9)	197,117 (44.7)	0.3
DPP-4i	205,550 (46.7)	203,469 (46.2)	0.9
TZD	48,775 (11.1)	46,710 (10.6)	1.5
GLP-1RA	36,903 (8.4)	34,033 (7.7)	2.4
Insulin	109,470 (24.8)	104,973 (23.8)	2.4
Anti-hypertensive therapy	308,944 (70.1)	304,417 (69.1)	2.2
Loop diuretics	38,852 (8.8)	37,785 (8.6)	0.9
Low ceiling diuretics	54,358 (12.3)	53,731 (12.2)	0.4
ACEi	97,176 (22.1)	97,019 (22.0)	0.1
ARBs	183,048 (41.5)	181,136 (41.1)	0.9
Statin therapy	288,674 (65.5)	286,787 (65.1)	0.9
Beta blockers	114,079 (25.9)	112,092 (25.4)	1.0
Aldosterone antagonists	14,215 (3.2)	13,998 (3.2)	0.3
Index year			
2012	20 (0.0)	109 (0.1)	2.6
2013	12,006 (5.5)	11,889 (5.4)	0.2
2014	64,187 (16.2)	63,126 (15.9)	0.7
2015	120,957 (30.8)	121,370 (30.9)	0.2
2016	206,590 (49.3)	205,948 (49.2)	0.3
2017	36,839 (20.7)	38,157 (21.4)	1.8

All values are n (%) unless otherwise stated; the denominator varies between each variable due to data availability for each database; * ≥ 1 hospitalization of ≥ 3 consecutive days during the year prior to index

*ACE* angiotensin converting enzyme, *ARB* angiotensin receptor blockers, *CKD* chronic kidney disease, *CV* cardiovascular, *DPP-4i* dipeptidyl peptidase-4 inhibitor, *GLP-1RA* glucagon-like peptide-1 receptor agonist, *oGLD* other glucose-lowering drug, *PAD* peripheral artery disease, *SGLT-2* sodium–glucose cotransporter-2, *std diff* standardized difference, *SU* sulfonylureas, *TZD* thiazolidinediones

The incidence rate (IR) for each outcome was assessed by treatment group as the number of events divided by the total number of person-years at risk. The time to first event was compared between groups using Cox proportional hazards models, presented as hazard ratios (HR; 95%CI) for each outcome separately by country. The primary analysis used an intent-to-treat (ITT) approach where patients were followed from the start of index treatment until either occurrence of the first outcome event or the censoring date (whichever came first), regardless of whether index treatment was discontinued. The HRs for each endpoint from each individual country were then pooled for an overall weighted summary [22], using random-effects models with inverse variance weighting for each country [23].

Analyses for each outcome were also stratified according to the presence of prior CVD [defined as history of myocardial infarction, unstable angina, heart failure, atrial fibrillation, stroke, percutaneous coronary intervention (PCI) or coronary artery bypass graft (CABG)]; patient age and sex; history of heart failure, chronic kidney disease (CKD), or cancer; baseline use of angiotensin-converting enzyme inhibitors (ACEi) or angiotensin receptor blockers (ARBs), β-blockers, high ceiling diuretics, aldosterone antagonists, insulin, sulphonylureas, and statins.

Sensitivity analyses were performed in order to evaluate the stability of the findings: data for the primary analysis were additionally adjusted for multiple covariates [age, gender, frailty (defined as at least one hospitalization of at least three consecutive days during the year prior to index), history of heart failure, history of myocardial infarction, atrial fibrillation history, hypertension (if available), obesity/BMI (if available), duration of diabetes (if available), and use of ACEi or ARBs, β-blockers, Ca^2+^-channel blockers, statins, loop diuretics and thiazide diuretics]; analyses were repeated using an on-treatment approach (follow-up censored at index treatment discontinuation).

Informed consent was not required, as the data were collected for clinical and administrative purposes and were analysed after de-identification. Analyses of data were conducted in accordance with local laws and regulations, and received approvals from Scientific/Ethics/Data Protection Committees in each country. Country-specific analyses were conducted by independent academic/statistical groups in each country. Meta-analyses were conducted by Statisticon AB, Uppsala (Sweden) and validated by independent academic statisticians at Saint Luke’s Mid America Heart Institute, Kansas City, Missouri (USA).

### Role of funding source

This analysis was overseen by the CVD-REAL (Comparative Effectiveness of Cardiovascular Outcomes in New Users of Sodium–Glucose Cotransporter-2 Inhibitors) Academic Scientific Committee, Study Group and Investigators, including members from the sponsor. The sponsor was involved in the design of the analysis, and the collection/interpretation of data. No payment was received by any author for writing this manuscript. The corresponding author and senior author had full access to all data, and vouch for the accuracy and completeness of data reported. All authors made the final decision to submit the manuscript.

## Results

### Study population

A total of 9,631,497 patients who newly initiated either SGLT-2i or oGLD treatment during the study period was identified (Additional file 1: Figure S1); 477,894 (5.0%) were new users of SGLT-2i and 9,153,603 (95.0%) were new users of oGLD. Prior to propensity score matching, the patients who initiated SGLT-2i were younger, had slightly less prevalent heart failure, stroke and CKD at baseline and greater use of statins, ACEis and low-ceiling diuretics (Additional file 1: Table S5). Patients initiated on SGLT-2i were also more likely to be receiving other glucose-lowering drugs at baseline (Additional file 1: Table S5).

Following propensity score matching, there were 440,599 new initiators of SGLT-2i and 440,599 new initiators of oGLDs (Additional file 1: Figure S1) and the baseline characteristics were well-balanced (Table 1). In both groups, the mean age was 58 years, 44% were women, and 31% had established CVD; 65% of patients received statins, 69% antihypertensive medications, and 76% metformin (Table 1). The distribution of specific SGLT-2i compounds initiated is shown in Additional file 1: Table S6; dapagliflozin contributed 60% of total exposure time, followed by canagliflozin (20%) and empagliflozin (14%), with other SGLT-2is providing smaller contributions. The distribution of oGLD by class is shown in Additional file 1: Table S7; the DPP-4i class contributed to 25% of the oGLD exposure time, followed by insulin (18%), SUs (18%), metformin (13%) and TZDs (11%); other classes (GLP-1 RA, acarbose and metiglinides) contributed < 10% each and made up the remainder.

**Fig. 1:**
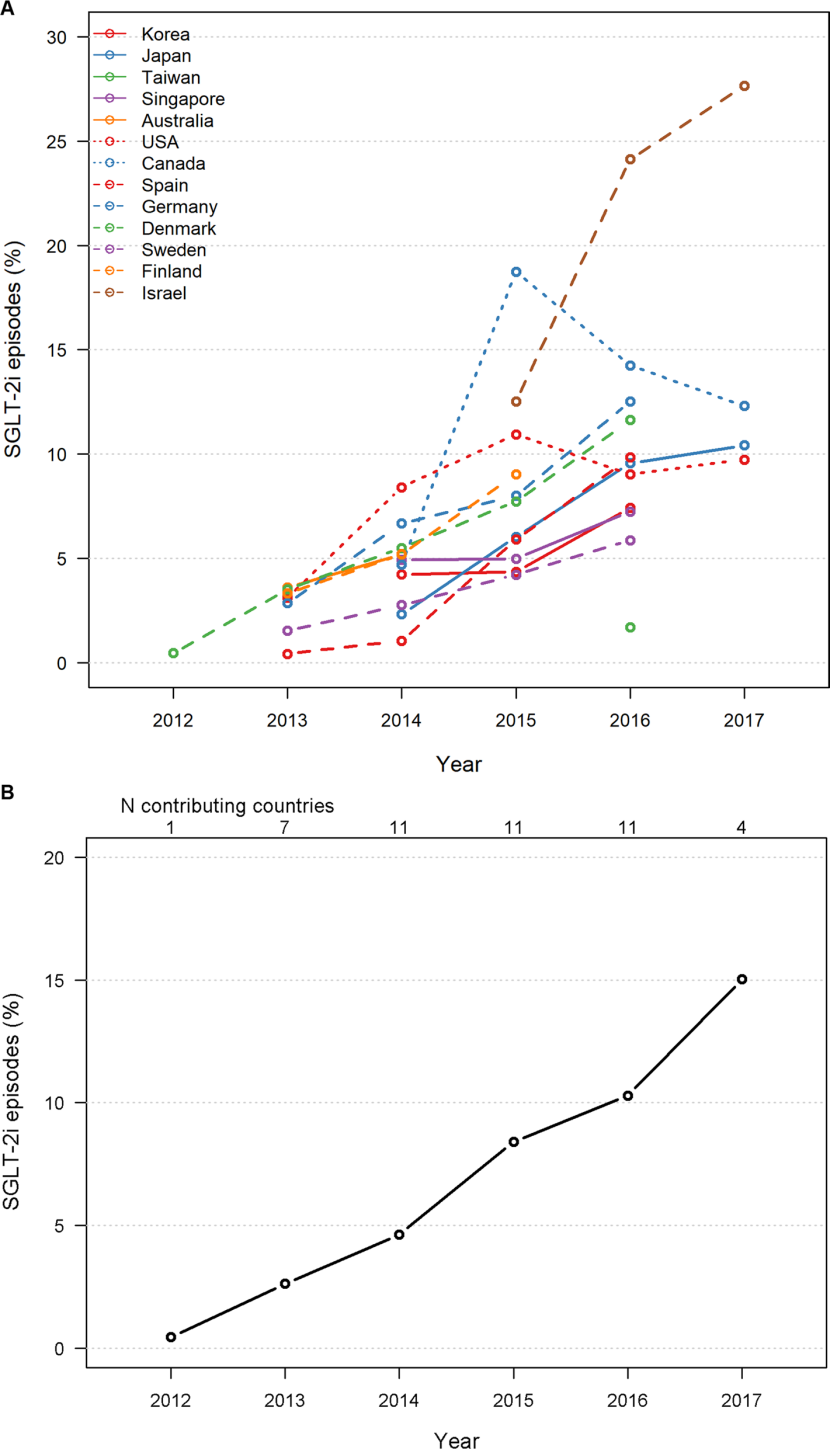
Trends in proportion of new user episodes which were SGLT-2i by **A** country and **B** overall in 2012–2017 (pre-propensity matching). *SGLT-2i* sodium–glucose cotransporter-2 inhibitor

Pre-propensity match, the proportion of new SGLT-2i episodes that were identified for each included country is shown in Fig. 1 as a function of time**.** For the countries included in this study, the proportion of new initiations that were SGLT-2i increased consistently over time; in North America, the proportion increased until 2015, with a slight decrease thereafter. When all countries included in this analysis were considered together, the proportion of new SGLT-2i initiation increased from around 3% of all new initiations in 2013, to about 15% in 2017 (Fig. 1).

### Outcomes

The mean follow-up time for the primary ITT analysis was 396 days for SGLT-2i and 406 days for oGLDs initiations (Additional file 1: Table S8).

**Fig. 2:**
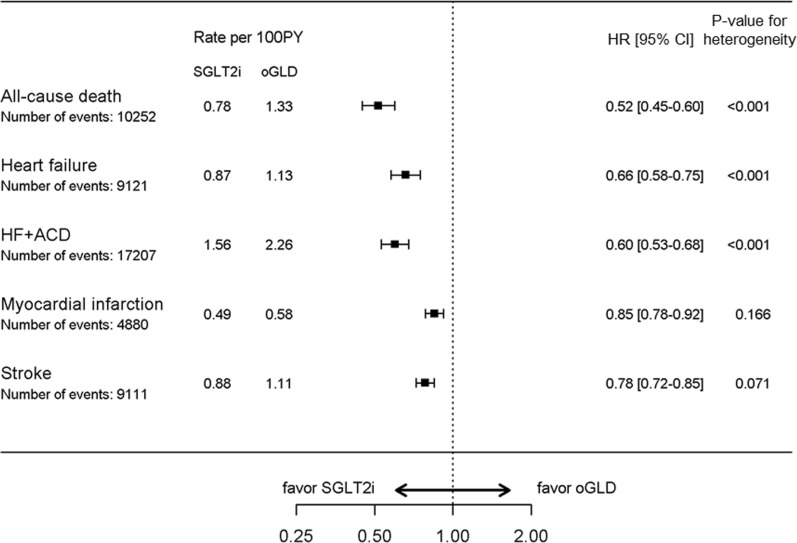
Pooled hazard ratios for the outcomes of hospitalization for heart failure, all-cause death, composite of hospitalization for heart failure or all-cause death, myocardial infarction, and stroke (Intent-to-treat analysis; unadjusted). *ACD* all-cause death, *HF* heart failure, *oGLD* other glucose-lowering drug, *PY* patient–years, *SGLT-2i* sodium–glucose cotransporter-2 inhibitor

During 914,208 patient-years of follow-up there were 9121 events of HHF (3913 in the SGLT-2i group and 5208 in the oGLD group; Additional file 1: Table S9). Initiation of SGLT-2i was associated with a lower risk of HHF (ITT-unadjusted pooled HR: 0.66, 95%CI 0.58–0.75; p < 0.001; Fig. 2). While there was heterogeneity, there were consistent associations between use of SGLT2i and lower risk of heart failure in all of the 13 countries (Additional file 1: Figure S2A).

During 968,452 patient-years of follow-up, there were 10,252 events of all-cause death (3712 in the SGLT-2i group and 6540 in the oGLD group; Additional file 1: Table S9). Initiation of SGLT-2i was associated with a lower risk of all-cause death (ITT-unadjusted pooled HR: 0.52, 95%CI 0.45–0.60; p < 0.001; Fig. 2). While there was heterogeneity, there were consistent associations between use of SGLT2i and lower risk of all-cause death in all of the 13 countries (Additional file 1: Figure S2B).

For the composite outcome of HHF or all-cause death, there were 17,207 events (6932 in the SGLT-2i group and 10,275 in the oGLD group) over 898,869 patient-years of follow-up (Additional file 1: Table S9). Initiation of SGLT-2i was associated with a lower risk of the composite of HHF or all-cause death (ITT-unadjusted pooled HR: 0.60, 95%CI 0.53–0.68; p < 0.001; Fig. 2). While there was heterogeneity, there were consistent associations between use of SGLT2i and lower risk of HHF or all-cause death in all of the 13 countries (Additional file 1: Figure S2C).

For myocardial infarction, there were 4880 events (2203 in the SGLT-2i group and 2677 in the oGLD group) over 916,305 patient-years of follow-up (Additional file 1: Table S9): initiation of SGLT-2i was associated with a lower risk of myocardial infarction (ITT-unadjusted pooled HR: 0.85, 95%CI 0.78–0.92; p < 0.001; Fig. 2; Additional file 1: Figure S2D).

For stroke, there were 9111 events (3981 in the SGLT-2i group and 5130 in the oGLD group) over 913,571 patient-years of follow-up (Additional file 1: Table S9): initiation of SGLT-2i was associated with a lower risk of stroke (ITT-unadjusted pooled HR: 0.78, 95%CI 0.72–0.85; p < 0.001; Fig. 2; Additional file 1: Figure S2E). There was no evidence of treatment heterogeneity across countries for the outcomes of myocardial infarction or stroke (p for interaction > 0.07).

### Subgroup and sensitivity analyses

**Fig. 3:**
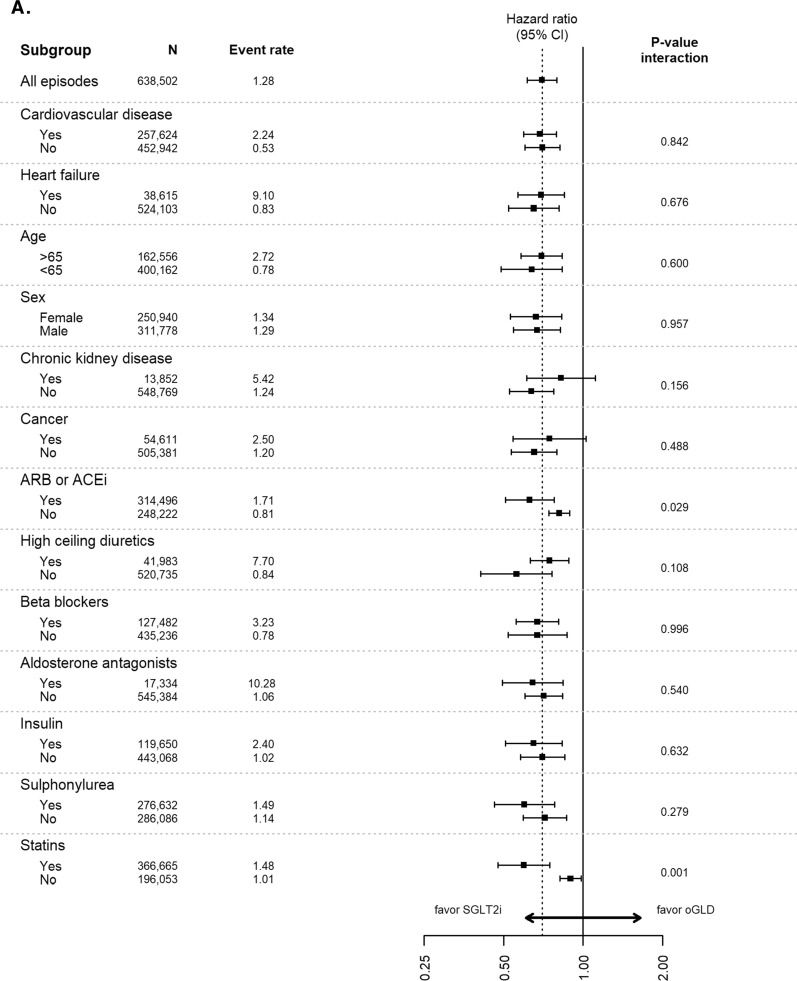

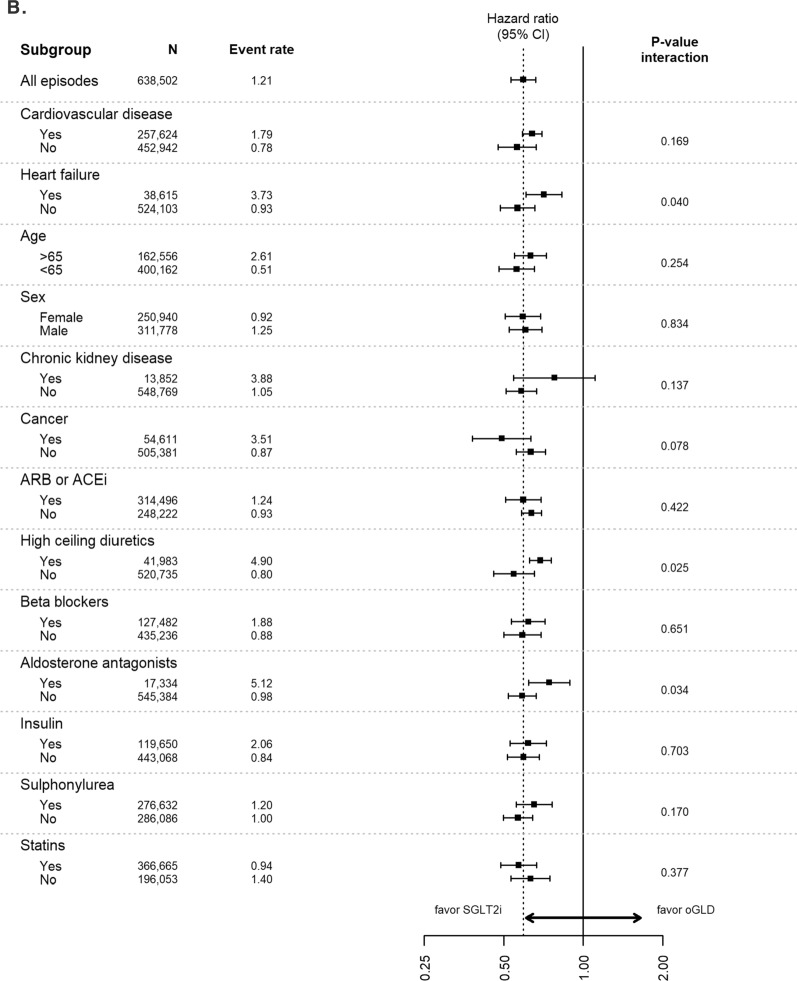

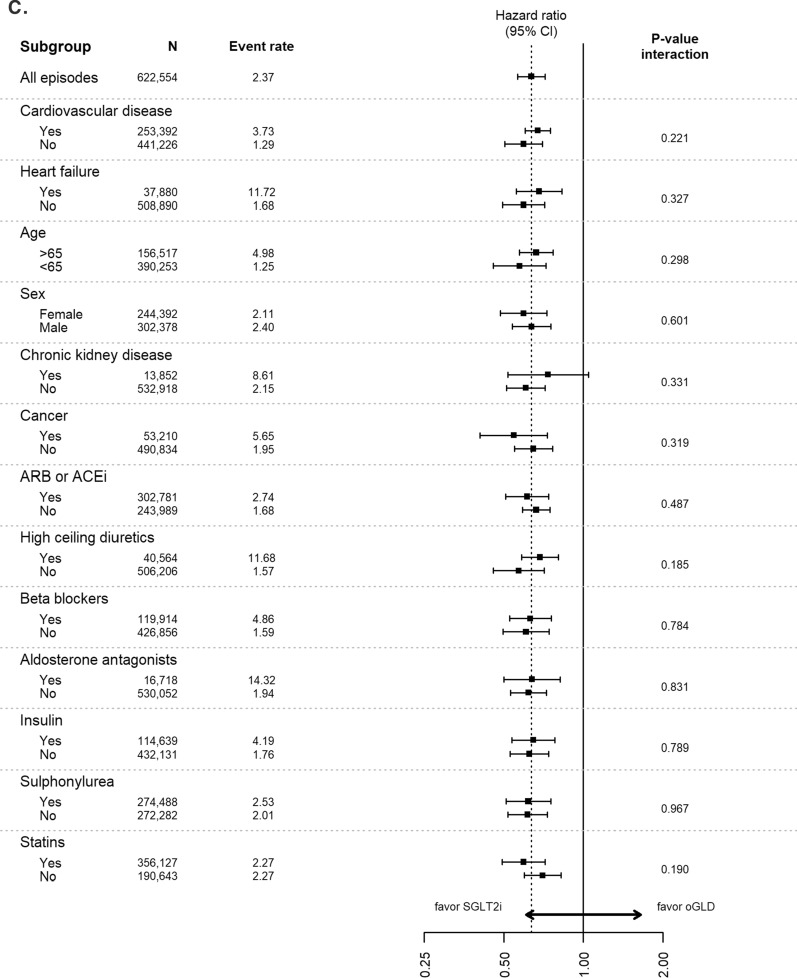

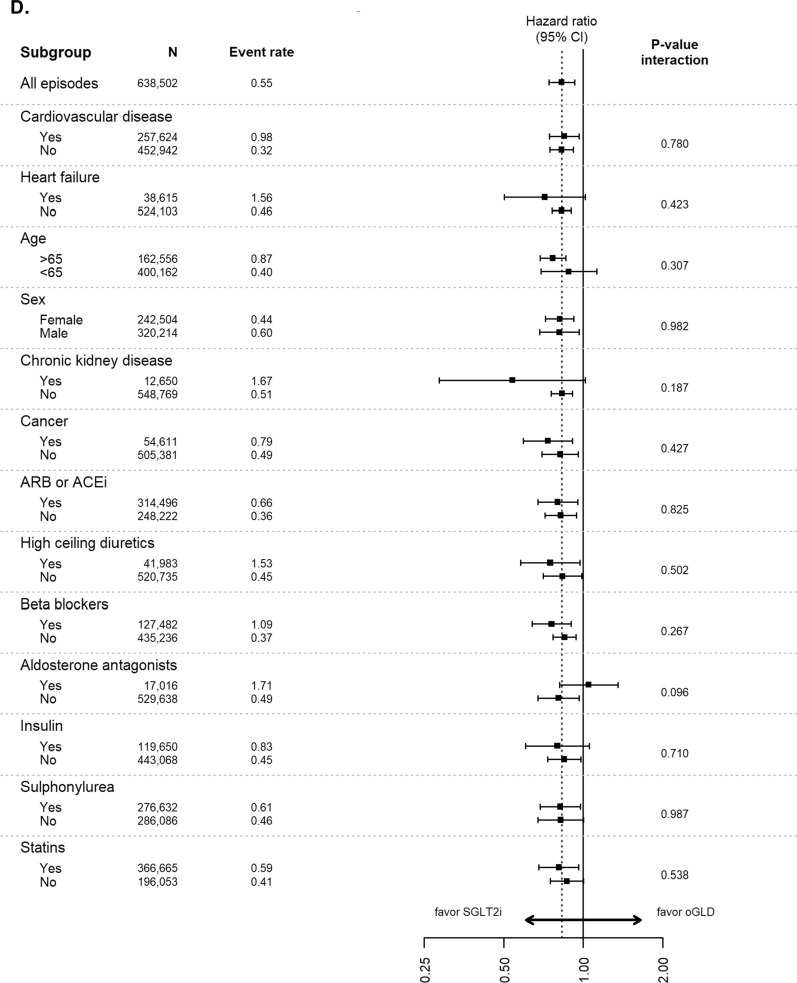

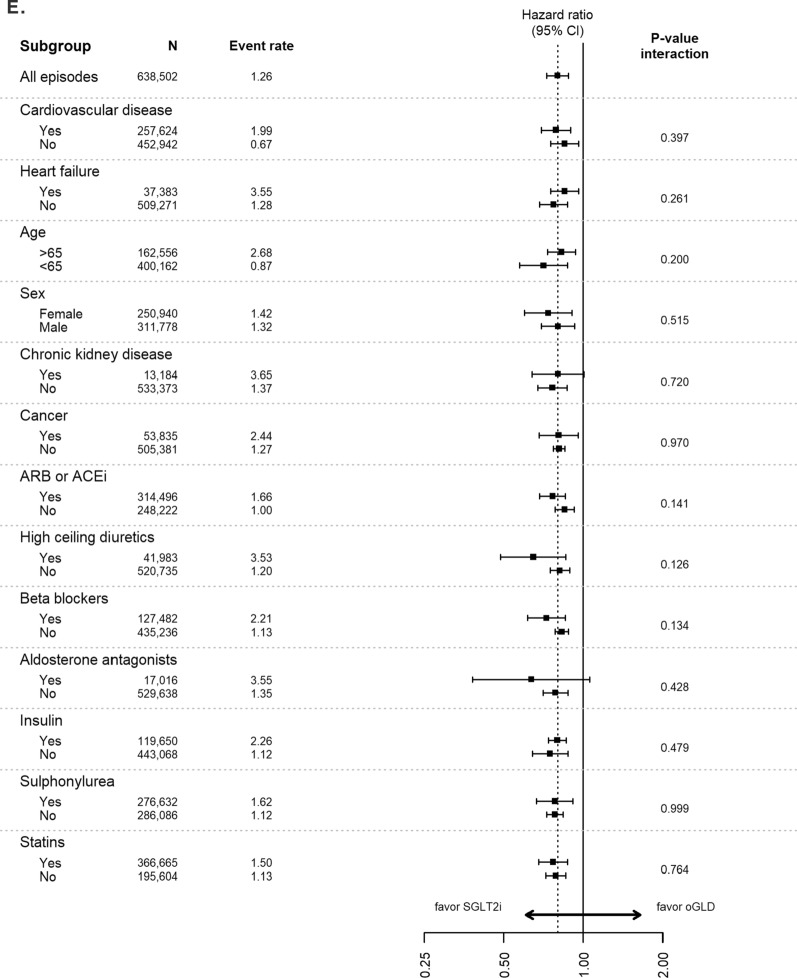
Pooled hazard ratios for **A** hospitalization for heart failure, **B** all-cause death, **C** composite of hospitalization for heart failure or all-cause death, **D** myocardial infarction, and **E** stroke stratified by subgroups (Intent-to-treat analysis; adjusted). Event rate, events per 100-person years; *ACE* angiotensin converting enzyme inhibitors, *ARB* angiotensin receptor blockers, *oGLD* other glucose-lowering drug, *SGLT-2i* sodium–glucose cotransporter-2 inhibitor

Analyses of data stratified by baseline characteristics continued to favor SGLT-2is over oGLD for the outcomes of HHF, all-cause death, composite of HHF or all-cause death, myocardial infarction and stroke across all subgroups, with very few significant interactions (Fig. 3).

Findings similar to the primary analyses were seen upon multivariable adjustment and in the on-treatment analyses (Additional file 1: Figures S3-S5).

## Discussion

In this latest update to the CVD-REAL study, that included nearly 10 million people from 13 countries, we report a number of notable findings. First, we show temporal trends in the prescribing of SGLT-2i therapies across three continents from 2012 to 2017, highlighting large global variations. Second, we show that in an analysis of data from a much larger number of countries and patients than previously reported, initiation of SGLT-2i therapy was associated with significantly lower risk of HHF, all-cause death, composite of HHF or all-cause death, myocardial infarction and stroke, compared with the initiation of oGLDs. Finally, we demonstrate that the clinical benefit of SGLT-2is compared to oGLDs was highly consistent across a number of prespecified subgroups, including age, sex, co-morbidities and use of various cardiovascular and anti-hyperglycemic agents. The results of this study, therefore, extend findings from prior CVD-REAL analyses and from the SGLT-2i inhibitor clinical trials in a broader, more ethnically diverse population from 13 countries.

Despite the significant cardiovascular benefits of SGLT-2i therapy reported in CVOTs and comparative effectiveness studies [10–12, 15, 16, 24–26], previous single country studies have shown that only a small proportion of people meeting the criteria for the CVOTs are prescribed these therapies [27, 28]. The DISCOVER study of 37 countries with 14,668 participants showed that, overall, the prescriptions for SGLT-2i were low during the study period 2014–2016, but also showed variations across continents [29]. In our study, assessment of the prescribing trends in the pre-matched patients from 2012 to 2017 demonstrated that, overall, use of SGLT-2i has increased since 2013, from around 3% of all new initiations in 2013 to about 15% in 2017; although this proportion remains low overall despite the evidence of the benefits of SGLT-2i. There were large variations between countries and over time, with the steepest increases in prescribing of SGLT-2i over this period seen in Canada and Israel. Recent data from the UK has also shown slow but steady increases in prescriptions for SGLT-2i as second-line therapy to metformin from 2014 to 2017 [30].

The populations of the SGLT-2i CVOTs differed in the proportions of patients with established CVD that were recruited. The EMPA-REG, CANVAS Programme and the DECLARE-TIMI 58 trials included 100%, 65.6% and 37.4%, respectively, people with type 2 diabetes and established atherosclerotic CVD, with the remainder having risk factors for CVD [10, 11, 31]. A meta-analysis of these three trials, with 34,322 patients and 3342 major cardiovascular events found that compared to placebo, SGLT-2i reduced major adverse cardiovascular events (cardiovascular death, myocardial infarction or stroke) by around 11% overall (HR 0.89, 95% CI 0.83–0.96), with benefit seen in those with established atherosclerotic CVD [14]. Similarly the meta-analysis also demonstrated a benefit with SGLT-2i against the risk of cardiovascular deaths or HHF (HR 0.77, 95% CI 0.71–0.84), and this benefit was seen across those with and without established CVD [14]. The results from this analysis of the CVD-REAL data, where the proportion of participants with established CVD was much lower at just over 30%, complement the findings from the CVOTs, and are similar to those seen in previous observational studies [24–26], thus further supporting the benefits of SGLT-2i in improving cardiovascular outcomes across a broad patient population. As one would expect, the absolute risks reductions are lower for those without versus with established CVD, yet the relative risk reductions are similar in both groups. The difference in the patient populations included in comparative-effectiveness studies and the CVOTs (where strict inclusion criteria are used), may explain some of the difference in magnitude of benefit seen between these studies.

There are a number of strengths in our study. This is the largest comparative effectiveness study of any glucose-lowering therapy to date with over 880,000 propensity-matched patients from a pooled population from 13 countries with different healthcare systems. Despite a lower cardiovascular risk population, we had a very large number of events in view of the size of the study cohort, allowing adequate power for multiple subgroup analyses. When considering the number of all-cause deaths there were 10,252 events in our study compared to 5216 in CVD-REAL 2; while in the CVOTs there were 463 events in EMPA-REG OUTCOME; 681 events in the CANVAS Programme; and 1099 in the DECLARE-TIMI 58 study [10–12, 15, 16]. This is a major strength of real-world comparative-effectiveness studies, as this large number of events in a broad population would not be feasible in a randomized controlled trial. As with other CVD-REAL studies, we found no geographic heterogeneity in outcomes from the 13 countries with the direction of the association for the benefits for SGLT-2i similar in all countries; and as some countries or regions favor specific agents (such as dapagliflozin in South Korea or canagliflozin the USA) this suggests generally similar effects for SGLT-2is contributing to this study.

These findings should be considered in light of some important limitations. Despite using robust statistical techniques including propensity score matching, the possibility of residual unmeasured confounding cannot be excluded. The data used in this analysis did not include important patientlevel data such as risk factor control, or comprehensive comorbidity data at baseline for some of the countries. In Japan and Singapore only in-hospital deaths were included. However, as most fatal events in these countries occur in hospital [20, 21], and the estimates for the outcomes were directionally similar to the other countries, where capture of the mortality data was more comprehensive, the results for all-cause death in these countries are consistent with our findings in other participating countries. The countries included in this study were all high-income, and therefore these findings may not be applicable to all patient populations. Safety of SGLT-2i versus oGLD was not assessed in our analysis; however, other randomized controlled trials and real-world studies have confirmed the relative safety of the SGLT-2i class [32]. While we included a large numbers of patients, the overall follow-up was relatively short (just over 12 months) given the relatively recent introduction of SGLT-2i. However, this is the longest length of follow-up across all previous CVD-REAL studies, and is similar to recently published comparative effectiveness studies with a smaller cohort of patients, with findings consistent with ours [24–26]. Longer term follow-up would be informative to evaluate the outcomes over time.

## Conclusion

The initiation of SGLT-2i as compared with other glucose-lowering drugs in routine clinical practice is associated with decreased risk of heart failure, all-cause death, myocardial infarction and stroke. The results of this large comparative effectiveness study with a broader, globally diverse population complement and extend the findings of completed randomized controlled trials.

## Supplementary Information

**Additional file 1.** CVD-REAL Investigator and Study Group.

## Data Availability

Data underlying the findings described in this manuscript may be obtained in accordance with AstraZeneca’s data sharing policy described at https://astrazenecagrouptrials.pharmacm.com/ST/Submission/Disclosure.

## Abbreviations

ACD: All-cause death
ACEi: Angiotensin-converting enzyme inhibitor
ARB: Angiotensin receptor blockers
CABG: Coronary artery bypass graft
CKD: Chronic kidney disease
CVD: Cardiovascular disease
HHF: Hospitalization for heart failure
HR: Hazard ratio
IR: Incidence rate
ITT: Intent-to-treat
MI: Myocardial infarction
oGLDs: Other glucose-lowering drugs
PCI: Percutaneous coronary intervention
SD: Standard deviation
SGLT-2i: Sodium–glucose cotransporter-2 inhibitor
