# Supplementary material for: Cardiovascular outcomes with sodium–glucose cotransporter-2 inhibitors vs other glucose-lowering drugs in 13 countries across three continents: analysis of CVD-REAL data
Source: Cardiovasc Diabetol. 2021 Jul 31;20:159. doi: 10.1186/s12933-021-01345-z (PMC8325810; doi:10.1186/s12933-021-01345-z)
Supplement: Supplementary file 1 — Additional file 1. CVD-REAL Investigator and Study Group. [file 12933_2021_1345_MOESM1_ESM.docx]

**Supplementary Materials**

**CVD-REAL Investigator and Study Group**

***Executive Scientific Committee (Academic Members and Investigators):***

**Mikhail Kosiborod, MD,** Saint Luke's Mid America Heart Institute and University of Missouri-Kansas City, Kansas City, MO, United States; **Matthew A. Cavender, MD MPH,** University of North Carolina, Chapel Hill, NC, United States; **John P. Wilding MD, PhD,** University of Liverpool, Liverpool, United; Kingdom; **Kamlesh Khunti, MD PhD,** University of Leicester, Leicester, United Kingdom; **Anna Norhammar, MD,** Cardiology Unit, Department of Medicine, Karolinska Institutet, Stockhom, Sweden; **Kåre Birkeland, MD PhD,** Department of Endocrinology, University of Oslo, Norway; **Marit Eika Jørgensen, MD PhD, Cand.med**, Steno Diabetes Center, Gentofte, Denmark; **Reinhard W. Holl MD PhD,** Institute of Epidemiology, University of Ulm, Ulm, Germany; **Carolyn SP Lam, MD,** National Heart Centre, Singapore and SingHealth Duke-NUS, Singapore.

***Extended publications committee***

**Hanne Løvdal Gulseth, MD, PhD,** Department of Endocrinology, Oslo University Hospital, Aker, Norway; **Bendix Carstensen, PhD,** Diabetes Center, Gentofte, Denmark; **Esther Bollow,** Institute of Epidemiology and Medical Biometry, University of Ulm, Ulm, Germany; **Josep Franch-Nadal, MD, PhD,** Researcher at the IDIAP - Unitad de Suport a la Recerca - Jordi Gol Fundation - EAP Rabal Sud, Barcelona, Spain - Catalunya Registry; **Luis Alberto García Rodríguez, MD,** CEIFE - Centro Español de Investigación Farmacoepidemiológica, Madrid, Spain; **Avraham Karasik, MD PhD**, Institute of Endocrinology, Tel Aviv University, Ramat Aviv, Israel - Maccabi Healthcare Services; **Navdeep Tangri MD PhD,** University of Manitoba, Winnipeg MB, Canada- Manitoba Centre for Health Policy (MCHP); **Shun Kohsaka MD,** Keio University School of Medicine, Tokyo, Japan; **Dae Jung Kim MD,** Professor at Ajou University School of Medicine, Korea; **Jonathan Shaw MD,** Professor, Baker IDI Heart and Diabetes Institute – Australia; **Suzanne Arnold, MD, MHA.** Research Assistant Professor, University of Missouri–Kansas City School of Medicine, Kansas City, MO; **Su-Yen Goh MD,** Singapore General Hospital, Singapore; **Chern-En Chiang MD**, Taipei Veterans General Hospital, Taipei, Taiwan; **Johan G Eriksson**, University of Helsinki and Helsinki University Hospital, Helsinki, Finland; **Francesco Zaccardi MD PhD**, University of Leicester, Leicester, United Kingdom

***Executive Scientific Committee (AstraZeneca Members):***

**Peter Fenici, MD, PhD**, Global Medical Affairs Senior Leader, AZ, Academy House, Cambridge, United Kingdom (Study Leader); **Johan Bodegård, MD PhD**, Medical Evidence Scientific Lead Nordics/Baltic, AZ Oslo, Norway; **Hungta Chen PhD,**  Principal Statistician, Biometrics and Information Sciences, AZ Gaithersburg, MD, USA; **Filip Surmont MD,** Medical Director CVMD International, AZ Luton, United Kingdom; **Rachel Kendrick, PhD**, Publications Lead, AZ Cambridge, United Kingdom; **Wesley Belli, PhD**, Publications Lead, AZ Gaithersburg, MD, USA;

***Study Core Team (AstraZeneca Members)*:**

**Eric T. Wittbrodt, PharmD**, **MPH**, HEOR Director, AZ Wilmington, DE, United States; **Matthias Saathoff, Dr**, Clinical Project Leader, ERP, AZ, Wedel, Germany; **Yusuke Noguchi**, BS, MBA, Associate Medical Affairs Manager in AZKK, Japan, **Donna Tan**, Brand Manager, COM, AZ Singapore, **Maro Williams**, Senior Medical Manager, EMA, AZ Australia, **Hye Won Lee**, Medical Science Liaison, COO, AZ South Korea, **Maya Greenbloom**, Manager Medical Liaison, Medical Affairs, AZ Israel, **Oksana Kaidanovich-Beilin**, Medical Evidence Lead, COO, AZ Canada; **Karolina Andersson-Sundell, MSc** PhD, Assoc .Dir. Epidemiology, MEOR, AZ Gothenburg, Sweden;.

***External Investigators and Analysts***:

**Khung Keong Yeo MBBS,** National Heart Centre, Singapore; **Yong Mong Bee MBBS**, Singapore General Hospital, Singapore; **Joan Khoo MBBS**, Changi General Hospital, Singapore; **Agnes Koong MBBS**, SingHealth Polyclinics, Singapore; **Yee How Lau**, Singapore Cardiac Data Bank, Singapore; **Fei Gao**, National Heart Centre, Singapore; **Wee Boon Tan MBBS**, Singapore General Hospital, Singapore; **Hanis Abdul Kadir BSc**, Singapore General Hospital, Singapore; **Kyoung Hwa Ha, PhD,** Department of Endocrinology and Metabolism, Ajou University School of Medicine, Republic of Korea; **Jinhee Lee, MS,** Department of Endocrinology and Metabolism, Ajou University School of Medicine, Republic of Korea; **Gabriel Chodick, PhD**, Faculty of Medicine, Tel Aviv University, Medical division, Maccabi Healthcare Services, Israel; **Cheli Melzer Cohen, MSc**, Medical division, Maccabi Healthcare Services, Israel; **Reid Whitlock, BEd, BSc, MSc**, Biostatistician, Seven Oaks Hospital Chronic Disease Innovation Centre, Canada; **Lucia Cea Soriano, PharmD, PhD**, CEIFE – Centro Español de Investigación Farmacoepidemiológica; **Oscar Fernándex Cantero**, CEIFE – Centro Español de Investigación Farmacoepidemiológica; **Jordan A. Menzin, BA**, Chief Technology Officer, Boston Health Economics, Inc, Waltham, MA; **Matthew Guthrie, MA,** Analyst II, Boston Health Economics, Inc, Waltham, MA; **Jennie Ilomaki PhD,** Monash University, Australia; **Dianna Magliano**, **PhD**, Monash University, Australia; **Fabian Hoti**, StatFinn & EPID Research, Espoo, Finland; **Solomon Christopher,** StatFinn & EPID Research, Espoo, Finland; **Minna Vehkala,** StatFinn & EPID Research, Espoo, Finland

**Data Sources**

Data from Finland utilised the DAHLIA study database, which includes T2D patient information from four linked national Finnish registries with full coverage of the population: the Finnish Prescription Register for years 1998 to 2015, covering all filled drug prescriptions using ATC-codes; the Finnish Cause of Death Registry for years until 2015; the Finnish Care Register for Health Care covering all open patient clinic visit diagnoses and all hospital discharge diagnoses for the years 1996 to 2015; and the Finnish Register for Primary Health Care visits for the years 2011-2015. Diagnoses are recorded according to the ICD-10 and the NOMESCO Classification of Surgical Procedure (NCSP) codes. Data linkage was performed by Finnish Social Insurance Institution based on the study identification numbers provided by the Prescription Register.

**Table S1. ICD and Read codes for Type 2 diabetes, Type 1 diabetes and gestational diabetes**

| **Type 2 diabetes** | |
| --- | --- |
| ***ICD codes*** | |
| ICD-9 250.X0, 250.X2 | |
| ICD-10 codes E11 and 024.1 | |
| ***Read codes*** | |
| 66A4.00 | Diabetic on oral treatment |
| 66Ao.00 | Diabetes type 2 review |
| 66At100 | Type II diabetic dietary review |
| 66At111 | Type 2 diabetic dietary review |
| 66AV.00 | Diabetic on insulin and oral treatment |
| C100100 | Diabetes mellitus, adult onset, no mention of complication |
| C100111 | Maturity onset diabetes |
| C100112 | Non-insulin dependent diabetes mellitus |
| C101100 | Diabetes mellitus, adult onset, with ketoacidosis |
| C102100 | Diabetes mellitus, adult onset, with hyperosmolar coma |
| C103100 | Diabetes mellitus, adult onset, with ketoacidotic coma |
| C104100 | Diabetes mellitus, adult onset, with renal manifestation |
| C105100 | Diabetes mellitus, adult onset, + ophthalmic manifestation |
| C106100 | Diabetes mellitus, adult onset, + neurological manifestation |
| C107100 | Diabetes mellitus, adult, + peripheral circulatory disorder |
| C107200 | Diabetes mellitus, adult with gangrene |
| C107400 | NIDDM with peripheral circulatory disorder |
| C109.00 | Non-insulin dependent diabetes mellitus |
| C109.11 | NIDDM - Non-insulin dependent diabetes mellitus |
| C109.12 | Type 2 diabetes mellitus |
| C109.13 | Type II diabetes mellitus |
| C109000 | Non-insulin-dependent diabetes mellitus with renal comps |
| C109011 | Type II diabetes mellitus with renal complications |
| C109012 | Type 2 diabetes mellitus with renal complications |
| C109100 | Non-insulin-dependent diabetes mellitus with ophthalm comps |
| C109111 | Type II diabetes mellitus with ophthalmic complications |
| C109112 | Type 2 diabetes mellitus with ophthalmic complications |
| C109200 | Non-insulin-dependent diabetes mellitus with neuro comps |
| C109211 | Type II diabetes mellitus with neurological complications |
| C109212 | Type 2 diabetes mellitus with neurological complications |
| C109300 | Non-insulin-dependent diabetes mellitus with multiple comps |
| C109400 | Non-insulin dependent diabetes mellitus with ulcer |
| C109411 | Type II diabetes mellitus with ulcer |
| C109412 | Type 2 diabetes mellitus with ulcer |
| C109500 | Non-insulin dependent diabetes mellitus with gangrene |
| C109511 | Type II diabetes mellitus with gangrene |
| C109512 | Type 2 diabetes mellitus with gangrene |
| C109600 | Non-insulin-dependent diabetes mellitus with retinopathy |
| C109611 | Type II diabetes mellitus with retinopathy |
| C109612 | Type 2 diabetes mellitus with retinopathy |
| C109700 | Non-insulin dependent diabetes mellitus - poor control |
| C109711 | Type II diabetes mellitus - poor control |
| C109712 | Type 2 diabetes mellitus - poor control |
| C109900 | Non-insulin-dependent diabetes mellitus without complication |
| C109A00 | Non-insulin dependent diabetes mellitus with mononeuropathy |
| C109A11 | Type II diabetes mellitus with mononeuropathy |
| C109B00 | Non-insulin dependent diabetes mellitus with polyneuropathy |
| C109B11 | Type II diabetes mellitus with polyneuropathy |
| C109C00 | Non-insulin dependent diabetes mellitus with nephropathy |
| C109C11 | Type II diabetes mellitus with nephropathy |
| C109C12 | Type 2 diabetes mellitus with nephropathy |
| C109D00 | Non-insulin dependent diabetes mellitus with hypoglyca coma |
| C109D11 | Type II diabetes mellitus with hypoglycaemic coma |
| C109D12 | Type 2 diabetes mellitus with hypoglycaemic coma |
| C109E00 | Non-insulin depend diabetes mellitus with diabetic cataract |
| C109E11 | Type II diabetes mellitus with diabetic cataract |
| C109E12 | Type 2 diabetes mellitus with diabetic cataract |
| C109F00 | Non-insulin-dependent d m with peripheral angiopath |
| C109F11 | Type II diabetes mellitus with peripheral angiopathy |
| C109F12 | Type 2 diabetes mellitus with peripheral angiopathy |
| C109G00 | Non-insulin dependent diabetes mellitus with arthropathy |
| C109G11 | Type II diabetes mellitus with arthropathy |
| C109G12 | Type 2 diabetes mellitus with arthropathy |
| C109H00 | Non-insulin dependent d m with neuropathic arthropathy |
| C109H11 | Type II diabetes mellitus with neuropathic arthropathy |
| C109H12 | Type 2 diabetes mellitus with neuropathic arthropathy |
| C109J00 | Insulin treated Type 2 diabetes mellitus |
| C109J11 | Insulin treated non-insulin dependent diabetes mellitus |
| C109J12 | Insulin treated Type II diabetes mellitus |
| C109K00 | Hyperosmolar non-ketotic state in type 2 diabetes mellitus |
| C10C.11 | Maturity onset diabetes in youth |
| C10D.00 | Diabetes mellitus autosomal dominant type 2 |
| C10D.11 | Maturity onset diabetes in youth type 2 |
| C10F.00 | Type 2 diabetes mellitus |
| C10F.11 | Type II diabetes mellitus |
| C10F000 | Type 2 diabetes mellitus with renal complications |
| C10F011 | Type II diabetes mellitus with renal complications |
| C10F100 | Type 2 diabetes mellitus with ophthalmic complications |
| C10F111 | Type II diabetes mellitus with ophthalmic complications |
| C10F200 | Type 2 diabetes mellitus with neurological complications |
| C10F211 | Type II diabetes mellitus with neurological complications |
| C10F300 | Type 2 diabetes mellitus with multiple complications |
| C10F311 | Type II diabetes mellitus with multiple complications |
| C10F400 | Type 2 diabetes mellitus with ulcer |
| C10F411 | Type II diabetes mellitus with ulcer |
| C10F500 | Type 2 diabetes mellitus with gangrene |
| C10F511 | Type II diabetes mellitus with gangrene |
| C10F600 | Type 2 diabetes mellitus with retinopathy |
| C10F611 | Type II diabetes mellitus with retinopathy |
| C10F700 | Type 2 diabetes mellitus - poor control |
| C10F711 | Type II diabetes mellitus - poor control |
| C10F900 | Type 2 diabetes mellitus without complication |
| C10F911 | Type II diabetes mellitus without complication |
| C10FA00 | Type 2 diabetes mellitus with mononeuropathy |
| C10FA11 | Type II diabetes mellitus with mononeuropathy |
| C10FB00 | Type 2 diabetes mellitus with polyneuropathy |
| C10FB11 | Type II diabetes mellitus with polyneuropathy |
| C10FC00 | Type 2 diabetes mellitus with nephropathy |
| C10FC11 | Type II diabetes mellitus with nephropathy |
| C10FD00 | Type 2 diabetes mellitus with hypoglycaemic coma |
| C10FD11 | Type II diabetes mellitus with hypoglycaemic coma |
| C10FE00 | Type 2 diabetes mellitus with diabetic cataract |
| C10FE11 | Type II diabetes mellitus with diabetic cataract |
| C10FF00 | Type 2 diabetes mellitus with peripheral angiopathy |
| C10FF11 | Type II diabetes mellitus with peripheral angiopathy |
| C10FG00 | Type 2 diabetes mellitus with arthropathy |
| C10FG11 | Type II diabetes mellitus with arthropathy |
| C10FH00 | Type 2 diabetes mellitus with neuropathic arthropathy |
| C10FJ00 | Insulin treated Type 2 diabetes mellitus |
| C10FJ11 | Insulin treated Type II diabetes mellitus |
| C10FK00 | Hyperosmolar non-ketotic state in type 2 diabetes mellitus |
| C10FL00 | Type 2 diabetes mellitus with persistent proteinuria |
| C10FL11 | Type II diabetes mellitus with persistent proteinuria |
| C10FM00 | Type 2 diabetes mellitus with persistent microalbuminuria |
| C10FM11 | Type II diabetes mellitus with persistent microalbuminuria |
| C10FN00 | Type 2 diabetes mellitus with ketoacidosis |
| C10FP00 | Type 2 diabetes mellitus with ketoacidotic coma |
| C10FQ00 | Type 2 diabetes mellitus with exudative maculopathy |
| C10FR00 | Type 2 diabetes mellitus with gastroparesis |
| C10K.00 | Type A insulin resistance |
| C10K000 | Type A insulin resistance without complication |
| C10z100 | Diabetes mellitus, adult onset, + unspecified complication |
| L180600 | Pre-existing diabetes mellitus, non-insulin-dependent |
| **Type 1 diabetes** | |
| ***ICD codes*** | |
| ICD-9 codes 250.x1, 250.X3 | |
| ICD-10 codes E10 and O24 | |
| ***Read codes*** | |
| 66An.00 | Diabetes type 1 review |
| C108D11 | Type I diabetes mellitus with nephropathy |
| 66An.00 | Diabetes type 1 review |
| 66At000 | Type I diabetic dietary review |
| 66At011 | Type 1 diabetic dietary review |
| C100011 | Insulin dependent diabetes mellitus |
| C101000 | Diabetes mellitus, juvenile type, with ketoacidosis |
| C102000 | Diabetes mellitus, juvenile type, with hyperosmolar coma |
| C103000 | Diabetes mellitus, juvenile type, with ketoacidotic coma |
| C104000 | Diabetes mellitus, juvenile type, with renal manifestation |
| C105000 | Diabetes mellitus, juvenile type, + ophthalmic manifestation |
| C106000 | Diabetes mellitus, juvenile, + neurological manifestation |
| C107000 | Diabetes mellitus, juvenile +peripheral circulatory disorder |
| C107300 | IDDM with peripheral circulatory disorder |
| C108.00 | Insulin dependent diabetes mellitus |
| C108.11 | IDDM-Insulin dependent diabetes mellitus |
| C108.12 | Type 1 diabetes mellitus |
| C108.13 | Type I diabetes mellitus |
| C108000 | Insulin-dependent diabetes mellitus with renal complications |
| C108011 | Type I diabetes mellitus with renal complications |
| C108012 | Type 1 diabetes mellitus with renal complications |
| C108100 | Insulin-dependent diabetes mellitus with ophthalmic comps |
| C108112 | Type 1 diabetes mellitus with ophthalmic complications |
| C108200 | Insulin-dependent diabetes mellitus with neurological comps |
| C108211 | Type I diabetes mellitus with neurological complications |
| C108212 | Type 1 diabetes mellitus with neurological complications |
| C108300 | Insulin dependent diabetes mellitus with multiple complicatn |
| C108400 | Unstable insulin dependent diabetes mellitus |
| C108411 | Unstable type I diabetes mellitus |
| C108412 | Unstable type 1 diabetes mellitus |
| C108500 | Insulin dependent diabetes mellitus with ulcer |
| C108511 | Type I diabetes mellitus with ulcer |
| C108512 | Type 1 diabetes mellitus with ulcer |
| C108600 | Insulin dependent diabetes mellitus with gangrene |
| C108700 | Insulin dependent diabetes mellitus with retinopathy |
| C108711 | Type I diabetes mellitus with retinopathy |
| C108712 | Type 1 diabetes mellitus with retinopathy |
| C108800 | Insulin dependent diabetes mellitus - poor control |
| C108811 | Type I diabetes mellitus - poor control |
| C108812 | Type 1 diabetes mellitus - poor control |
| C108900 | Insulin dependent diabetes maturity onset |
| C108911 | Type I diabetes mellitus maturity onset |
| C108912 | Type 1 diabetes mellitus maturity onset |
| C108A00 | Insulin-dependent diabetes without complication |
| C108A11 | Type I diabetes mellitus without complication |
| C108B00 | Insulin dependent diabetes mellitus with mononeuropathy |
| C108B11 | Type I diabetes mellitus with mononeuropathy |
| C108C00 | Insulin dependent diabetes mellitus with polyneuropathy |
| C108D00 | Insulin dependent diabetes mellitus with nephropathy |
| C108D11 | Type I diabetes mellitus with nephropathy |
| C108E00 | Insulin dependent diabetes mellitus with hypoglycaemic coma |
| C108E11 | Type I diabetes mellitus with hypoglycaemic coma |
| C108E12 | Type 1 diabetes mellitus with hypoglycaemic coma |
| C108F00 | Insulin dependent diabetes mellitus with diabetic cataract |
| C108F11 | Type I diabetes mellitus with diabetic cataract |
| C108G00 | Insulin dependent diab mell with peripheral angiopathy |
| C108H00 | Insulin dependent diabetes mellitus with arthropathy |
| C108H11 | Type I diabetes mellitus with arthropathy |
| C108J00 | Insulin dependent diab mell with neuropathic arthropathy |
| C108J11 | Type I diabetes mellitus with neuropathic arthropathy |
| C108J12 | Type 1 diabetes mellitus with neuropathic arthropathy |
| C10C.12 | Maturity onset diabetes in youth type 1 |
| C10E.00 | Type 1 diabetes mellitus |
| C10E.11 | Type I diabetes mellitus |
| C10E.12 | Insulin dependent diabetes mellitus |
| C10E000 | Type 1 diabetes mellitus with renal complications |
| C10E012 | Insulin-dependent diabetes mellitus with renal complications |
| C10E100 | Type 1 diabetes mellitus with ophthalmic complications |
| C10E111 | Type I diabetes mellitus with ophthalmic complications |
| C10E112 | Insulin-dependent diabetes mellitus with ophthalmic comps |
| C10E200 | Type 1 diabetes mellitus with neurological complications |
| C10E212 | Insulin-dependent diabetes mellitus with neurological comps |
| C10E300 | Type 1 diabetes mellitus with multiple complications |
| C10E311 | Type I diabetes mellitus with multiple complications |
| C10E312 | Insulin dependent diabetes mellitus with multiple complicat |
| C10E400 | Unstable type 1 diabetes mellitus |
| C10E411 | Unstable type I diabetes mellitus |
| C10E412 | Unstable insulin dependent diabetes mellitus |
| C10E500 | Type 1 diabetes mellitus with ulcer |
| C10E511 | Type I diabetes mellitus with ulcer |
| C10E512 | Insulin dependent diabetes mellitus with ulcer |
| C10E600 | Type 1 diabetes mellitus with gangrene |
| C10E611 | Type I diabetes mellitus with gangrene |
| C10E700 | Type 1 diabetes mellitus with retinopathy |
| C10E711 | Type I diabetes mellitus with retinopathy |
| C10E712 | Insulin dependent diabetes mellitus with retinopathy |
| C10E800 | Type 1 diabetes mellitus - poor control |
| C10E812 | Insulin dependent diabetes mellitus - poor control |
| C10E900 | Type 1 diabetes mellitus maturity onset |
| C10E911 | Type I diabetes mellitus maturity onset |
| C10E912 | Insulin dependent diabetes maturity onset |
| C10EA00 | Type 1 diabetes mellitus without complication |
| C10EA11 | Type I diabetes mellitus without complication |
| C10EA12 | Insulin-dependent diabetes without complication |
| C10EB00 | Type 1 diabetes mellitus with mononeuropathy |
| C10EC00 | Type 1 diabetes mellitus with polyneuropathy |
| C10EC11 | Type I diabetes mellitus with polyneuropathy |
| C10EC12 | Insulin dependent diabetes mellitus with polyneuropathy |
| C10ED00 | Type 1 diabetes mellitus with nephropathy |
| C10ED12 | Insulin dependent diabetes mellitus with nephropathy |
| C10EE00 | Type 1 diabetes mellitus with hypoglycaemic coma |
| C10EE12 | Insulin dependent diabetes mellitus with hypoglycaemic coma |
| C10EF00 | Type 1 diabetes mellitus with diabetic cataract |
| C10EF12 | Insulin dependent diabetes mellitus with diabetic cataract |
| C10EG00 | Type 1 diabetes mellitus with peripheral angiopathy |
| C10EH00 | Type 1 diabetes mellitus with arthropathy |
| C10EJ00 | Type 1 diabetes mellitus with neuropathic arthropathy |
| C10EK00 | Type 1 diabetes mellitus with persistent proteinuria |
| C10EL00 | Type 1 diabetes mellitus with persistent microalbuminuria |
| C10EL11 | Type I diabetes mellitus with persistent microalbuminuria |
| C10EM00 | Type 1 diabetes mellitus with ketoacidosis |
| C10EM11 | Type I diabetes mellitus with ketoacidosis |
| C10EN00 | Type 1 diabetes mellitus with ketoacidotic coma |
| C10EN11 | Type I diabetes mellitus with ketoacidotic coma |
| C10EP00 | Type 1 diabetes mellitus with exudative maculopathy |
| C10EP11 | Type I diabetes mellitus with exudative maculopathy |
| C10EQ00 | Type 1 diabetes mellitus with gastroparesis |
| C10z000 | Diabetes mellitus, juvenile type, + unspecified complication |
| L180500 | Pre-existing diabetes mellitus, insulin-dependent |
| 8Hj3.00 | Referral to DAFNE diabetes structured education programme |
| 8Hj4.00 | Referral to DESMOND diabetes structured education programme |
| 8Hj5.00 | Referral to XPERT diabetes structured education programme |
| 8I82.00 | Did not complete DAFNE diabetes structured education program |
| 8I83.00 | Did not complete DESMOND diabetes structured educat program |
| 8I84.00 | Did not complete XPERT diabetes structured education program |
| 9NiC.00 | Did not attend DAFNE diabetes structured education programme |
| 9NiD.00 | Did not attend DESMOND diabetes structured education program |
| 9NiE.00 | Did not attend XPERT diabetes structured education programme |
| 9OLG.00 | Attended XPERT diabetes structured education programme |
| 9OLH.00 | Attended DAFNE diabetes structured education programme |
| 9OLJ.00 | DAFNE diabetes structured education programme completed |
| 9OLK.00 | DESMOND diabetes structured education programme completed |
| 9OLL.00 | XPERT diabetes structured education programme completed |
| **Gestational Diabetes** | |
| ***ICD codes*** | |
| ICD-9 codes 648.8 | |
| ICD-10:O24.4 | |
| ***Read codes*** | |
| ZC2CB00 | Dietary advice for gestational diabetes |
| ZV13F00 | [V]Personal history of gestational diabetes mellitus |
| L180900 | Gestational diabetes mellitus |
| L180811 | Gestational diabetes mellitus |
| 8CE0000 | Gestational diabetes information leaflet given |
| 66Ay.00 | Gestational diabetes mellitus annual review |
| 8CE0000 | Gestational diabetes information leaflet given |
| Q44B.00 | Syndrome of infant of mother with gestational diabetes |
| 66AX.00 | Diabetes: shared care in pregnancy - diabetol and obstet |
| 6761.00 | Diabetic pre-pregnancy counselling |
| L180.00 | Diabetes mellitus during pregnancy/childbirth/puerperium |
| L180000 | Diabetes mellitus - unspec whether in pregnancy/puerperium |
| L180100 | Diabetes mellitus during pregnancy - baby delivered |
| L180300 | Diabetes mellitus during pregnancy - baby not yet delivered |
| L180800 | Diabetes mellitus arising in pregnancy |
| L180z00 | Diabetes mellitus in pregnancy/childbirth/puerperium NOS |

**Table S2. Start and stop dates per country**

| **Country** | **Start** | **Stop** |
| --- | --- | --- |
| **South Korea** | 2014 – September | 2016 – December |
| **Japan** | 2014 – April | 2017 – July |
| **Singapore** | 2014 – September | 2017 – November |
| **Australia** | 2013 – December | 2014 – December |
| **USA** | 2013 – April | 2017 – September |
| **Canada** | 2014 – June | 2017 – March |
| **Denmark** | 2012 – December | 2016 – December |
| **Sweden** | 2013 – July | 2016 – December |
| **Spain** | 2013 – Spain | 2016 – December |
| **Germany** | 2013 – January | 2016 – December |
| **Israel** | 2015 – April | 2017 – June |
| **Taiwan** | 2016 – May | 2016 – December |
| **Finland** | 2013 – August | 2015 – December |

**Table S3. Hospital discharge diagnosis codes**

|  | **Definition** |
| --- | --- |
| Heart failure | Hospitalization with a main diagnosis of heart failure (ICD10: I50, or equivalent) |
| All-cause death | Date of death |
| Cardiovascular death | Death with CV underlying cause of death: ICD10: I, or equivalent |
| Myocardial infarction | Hospitalization with a main diagnosis of myocardial infarction (ICD10: I21, or equivalent) |
| Stroke | Hospitalization with a main diagnosis of stroke (ICD10: I60-I64, or equivalent) |
| Ischemic stroke | Hospitalization with a main diagnosis of ischemic stroke (ICD10: I63-I64, or equivalent) |

CV, cardiovascular

**Table S4. Variables included in the propensity score***

| Women |
| --- |
| Age in years |
| Frailty |
| CV history |
| Smoker |
| HbA1c >7% |
| BMI ≥30 kg/m^2^ |
| Duration of type 2 diabetes |
| eGFR <60 |
| Chronic kidney disease |
| Microvascular disease |
| Nephropathy |
| Peripheral neuropathy |
| Retinopathy |
| Acute myocardial infarction |
| Stroke |
| Heart failure |
| Unstable angina |
| Atrial fibrillation |
| Peripheral artery disease |
| Hypertension |
| Coronary revascularization |
| Coronary artery bypass graft |
| Percutaneous coronary intervention |
| Carotid intervention |
| Metformin |
| Sulfonylurea |
| GLP-1 receptor agonists |
| Meglitinide |
| Thiazolidinedione |
| Acarbose |
| Insulins |
| Short-acting |
| Intermediate-acting (isophane) |
| Premixed |
| Long-acting |
| Statin therapy |
| Anti-hypertensive therapy |
| P2Y12 inhibitors |
| Antiplatelets |
| Warfarin |
| Anticoagulants |
| Low dose aspirin |
| Beta-blocker |
| Loop diuretics |
| Low ceiling diuretics |
| Aldosterone antagonists |
| Weight loss drugs |
| Bariatric surgery |
| Index date |

CV, cardiovascular; dipeptidylpeptidase; eGFR, estimated glomerular filtration rate; GLP-1, glucagon-like peptide-1

*Baseline comorbidity information was not available in Australia; laboratory variables were only available in Israel, Singapore and Canada; BMI was only available in Japan, Israel, Singapore and Canada; treatment duration was only available in Japan and Israel.

**Table S5**. **Baseline characteristics for all 13 countries combined (pre-match)**

|  | **SGLT-2 inhibitor (N=477,894)** | **oGLD (N=9,153,603)** | **std diff** |
| --- | --- | --- | --- |
| **Age, years** | 57.9 (11.7) | 63.0 (12.9) | 41.7 % |
| **Women** | 210,807 (44.1) | 4,090,539 (44.7) | 1.2 % |
| **CV-history** | 146,813 (31.6) | 3,360,740 (37.8) | 12.9 % |
| **Myocardial infarction** | 21,773 (4.7) | 389,070 (4.4) | 1.5 % |
| **Unstable angina** | 24,861 (5.4) | 439,384 (4.9) | 1.9 % |
| **Heart failure** | 35,309 (7.6) | 856,951 (9.6) | 7.2 % |
| **Atrial fibrillation** | 19,433 (4.2) | 458,860 (5.2) | 4.6 % |
| **Stroke** | 51,633 (11.1) | 1,518,552 (17.1) | 17.2 % |
| **PAD** | 24,059 (5.2) | 555,212 (6.2) | 4.6 % |
| **Microvascular disease** | 238,231 (51.3) | 4,122,796 (46.3) | 10.0 % |
| **CKD** | 26,135 (5.6) | 870,594 (9.8) | 15.6 % |
| **Frailty* (yes)** | 40,339 (8.9) | 1,084,273 (12.5) | 11.5 % |
| **Metformin** | 371,489 (77.7) | 4,089,779 (44.7) | 72.1 % |
| **SU** | 226,351 (47.4) | 3,013,375 (32.9) | 29.8 % |
| **DPP-4i** | 235,147 (49.2) | 2,488,956 (27.2) | 46.5 % |
| **TZD** | 56,960 (11.9) | 687,003 (7.5) | 14.9 % |
| **GLP-1RA** | 42,334 (8.9) | 143,012 (1.6) | 33.3 % |
| **Insulin** | 123,337 (25.8) | 1,558,294 (17.0) | 21.5 % |
| **Anti hypertensive therapy** | 337,210 (70.6) | 6,104,116 (66.7) | 8.4 % |
| **Loop diuretics** | 41,339 (8.7) | 1,037,777 (11.3) | 9.0 % |
| **Low ceiling diuretics†** | 57,170 (12.0) | 794,409 (8.7) | 10.8 % |
| **ACE Inhibitors** | 104,170 (21.8) | 1,414,670 (15.5) | 16.3 % |
| **ARBs** | 202,558 (42.4) | 3,677,650 (40.2) | 4.5 % |
| **Statin therapy** | 316,349 (66.2) | 4,883,923 (53.4) | 26.4 % |
| **Beta blockers** | 126,598 (26.5) | 2,445,812 (26.7) | 0.5 % |
| **Aldosterone antagonists** | 15,693 (3.3) | 320,714 (3.5) | 1.2 % |
| **Index year** |  |  |  |
| **2012** | 20 (0.0) | 4,468 (0.2) | 5.6 % |
| **2013** | 12,024 (5.3) | 399,571 (13.3) | 28.1 % |
| **2014** | 64,655 (15.8) | 1,247,057 (21.1) | 13.7 % |
| **2015** | 123,910 (30.3) | 2,012,810 (35.3) | 10.8 % |
| **2016** | 234,035 (51.3) | 5,169,578 (58.9) | 15.3 % |
| **2017** | 43,250 (22.6) | 331,268 (14.8) | 20.1 % |

All values are n (%) unless otherwise stated; *≥1 hospitalization of ≥3 consecutive days during the year prior to index. †, thiazides

ACE, angiotensin converting enzyme; ARB, angiotensin receptor blockers; CKD, chronic kidney disease; CV, cardiovascular; DPP-4i, dipeptidyl peptidase-4 inhibitor; GLP-1RA, glucagon-like peptide-1receptor agonist; oGLD, other glucose-lowering drug; PAD, peripheral artery disease; SGLT-2i, sodium-glucose cotransporter-2 inhibitor; std Diff, standardized difference; SU, sulfonylureas; TZD, thiazolidinediones.

**Table S6. Distribution of follow-up time among different SGLT-2i agents, as a percentage of time within each country**

|  | **Dapagliflozin** | **Empagliflozin** | **Canagliflozin** | **Ipragliflozin** | **Luseogliflozin** | **Tofogliflozin** |
| --- | --- | --- | --- | --- | --- | --- |
| **South Korea** | 91.3 | 4.3 | 0.0 | 4.4 | 0.0 | 0.0 |
| **Japan** | 26.1 | 13.6 | 10.6 | 29.8 | 6.3 | 13.7 |
| **Taiwan** | 59.7 | 40.3 | 0.0 | 0.0 | 0.0 | 0.0 |
| **Singapore** | 68.5 | 17.9 | 13.7 | 0.0 | 0.0 | 0.0 |
| **Australia** | 80.8 | 0.0 | 19.2 | 0.0 | 0.0 | 0.0 |
| **USA** | 12.9 | 15.9 | 71.2 | 0.0 | 0.0 | 0.0 |
| **Canada** | 52.0 | 7.7 | 40.2 | 0.0 | 0.0 | 0.0 |
| **Spain** | 62.1 | 13.7 | 24.2 | 0.0 | 0.0 | 0.0 |
| **Germany** | 79.7 | 18.2 | 2.1 | 0.0 | 0.0 | 0.0 |
| **Denmark** | 79.9 | 17.5 | 2.7 | 0.0 | 0.0 | 0.0 |
| **Sweden** | 75.9 | 23.8 | 0.3 | 0.0 | 0.0 | 0.0 |
| **Finland** | 97.5 | 2.5 | 0.0 | 0.0 | 0.0 | 0.0 |
| **Israel** | 50.9 | 49.1 | 0.0 | 0.0 | 0.0 | 0.0 |
| **Total** | **60.4** | **14.1** | **20.1** | **3.7** | **0.5** | **1.1** |

**Table S7. Distribution of follow-up time for each oGLD class, as percentage of time within each country**

|  | **Metformin** | **SU** | **DPP-4i** | **TZD** | **GLP-1i** | **Insulin** | **Acarbose** | **Metiglinides** |
| --- | --- | --- | --- | --- | --- | --- | --- | --- |
| **South Korea** | 11.5 | 19.2 | 36.2 | 22.0 | 0.4 | 7.3 | 2.6 | 0.6 |
| **Japan** | 20.6 | 13.4 | 23.5 | 10.2 | 3.5 | 7.5 | 11.7 | 9.6 |
| **Taiwan** | 9.5 | 24.6 | 19.8 | 0.6 | 0.8 | 16.6 | 20.5 | 7.6 |
| **Singapore** | 11.3 | 8.8 | 26.7 | 0.0 | 0.4 | 36.9 | 15.8 | 0.1 |
| **Australia** | 26.5 | 15.5 | 30.7 | 1.4 | 6.9 | 18.0 | 1.0 | 0.0 |
| **USA** | 12.6 | 24.0 | 17.6 | 10.3 | 14.9 | 18.5 | 0.7 | 1.5 |
| **Canada** | 9.5 | 29.6 | 32.1 | 2.0 | 1.5 | 20.8 | 2.3 | 2.3 |
| **Spain** | 21.0 | 15.6 | 20.4 | 2.7 | 11.3 | 19.1 | 0.3 | 9.6 |
| **Germany** | 23.1 | 12.1 | 23.2 | 0.3 | 11.7 | 24.1 | 0.8 | 4.7 |
| **Denmark** | 10.5 | 12.8 | 20.1 | 0.2 | 14.8 | 41.5 | 0.1 | 0.0 |
| **Sweden** | 8.4 | 12.2 | 16.9 | 0.9 | 13.2 | 44.1 | 0.3 | 4.1 |
| **Finland** | 5.0 | 5.6 | 14.2 | 6.0 | 23.0 | 43.8 | 0.0 | 2.2 |
| **Israel** | 5.3 | 12.4 | 18.9 | 11.4 | 19.6 | 17.1 | 1.8 | 13.5 |
| **Total** | **13.3** | **18.0** | **25.4** | **10.9** | **8.2** | **18.2** | **3.0** | **3.0** |

DPP-4i, dipeptyl peptidase-4 inhibitor; GLP-1i, glucagon-like peptide-1 inhibitor; oGLD, other glucose-lowering drug; SU, sulfonylurea; TZD, thiazolidinediones

**Table S8. Mean follow-up times (days) for the SGLT-2i and oGLD groups for the intent-to-treat and on-treatment analyses**

|  | **On treatment** | | **ITT** | |
| --- | --- | --- | --- | --- |
|  | **SGLT-2i** | **oGLD** | **SGLT-2i** | **oGLD** |
| **Korea** | 216 | 228 | 348 | 373 |
| **Japan** | 287 | 247 | 375 | 378 |
| **Taiwan** | 110 | 115 | 144 | 144 |
| **Singapore** | 319 | 361 | 405 | 464 |
| **Australia** | 127 | 116 | 652 | 643 |
| **USA** | 299 | 269 | 500 | 498 |
| **Canada** | 299 | 242 | 463 | 459 |
| **Spain** |  |  | 333 | 337 |
| **Germany** | 364 | 399 | 513 | 515 |
| **Denmark** | 393 | 411 | 512 | 505 |
| **Sweden** | 329 | 342 | 447 | 462 |
| **Finland** | 254 | 320 | 340 | 367 |
| **Israel** | 219 | 177 | 364 | 352 |
| **Total** | **243** | **240** | **396** | **406** |

ITT, intent-to-treat; oGLD, other glucose-lowering drug; SGLT-2i, Sodium-glucose cotransporter-2 inhibitor

**Table S9. Events and event rates (per 100 patient-years) for each outcome per country (ITT, unadjusted)**

|  |  | **HHF** | | **ACD** | | **HHF or ACD** | | **MI** | | **Stroke** | |
| --- | --- | --- | --- | --- | --- | --- | --- | --- | --- | --- | --- |
|  | **N** | **SGLT-2** | **oGLD** | **SGLT-2** | **oGLD** | **SGLT-2** | **oGLD** | **SGLT-2** | **oGLD** | **SGLT-2** | **oGLD** |
| **Korea** | 336644 | 2310 (1.45) | 2839 (1.67) | 1386 (0.86) | 2059 (1.20) | 3451 (2.17) | 4539 (2.66) | 821 (0.51) | 1080 (0.63) | 2608 (1.64) | 3364 (1.98) |
| **Japan** | 67780 | 242 (0.70) | 323 (0.93) | 200 (0.57) | 357 (1.02) | 419 (1.21) | 642 (1.84) | 39 (0.11) | 52 (0.15) | 108 (0.31) | 164 (0.47) |
| **Taiwan** | 68754 | 28 (0.21) | 77 (0.57) | 142 (1.05) | 263 (1.94) | 169 (1.25) | 333 (2.46) | 24 (0.18) | 57 (0.42) | 78 (0.58) | 118 (0.87) |
| **Singapore** | 2726 | 24 (1.60) | 43 (2.55) | 14 (0.93) | 22 (1.27) | 33 (2.21) | 60 (3.56) | 29 (1.95) | 41 (2.42) | 8 (0.53) | 26 (1.53) |
| **Australia** | 27442 |  |  | 177 (0.72) | 541 (2.24) |  |  |  |  |  |  |
| **USA** | 189420 | 231 (0.18) | 404 (0.31) | 564 (0.43) | 901 (0.70) | 769 (0.59) | 1244 (0.97) | 609 (0.47) | 685 (0.53) | 412 (0.32) | 541 (0.42) |
| **Canada** | 16064 | 25 (0.25) | 63 (0.63) | 94 (0.92) | 167 (1.65) | 115 (1.13) | 216 (2.15) | 53 (0.52) | 75 (0.75) | 20 (0.20) | 25 (0.25) |
| **Spain** | 25834 | 164 (1.41) | 244 (2.08) | 112 (0.95) | 270 (2.26) | 263 (2.26) | 485 (4.13) | 56 (0.48) | 54 (0.45) | 96 (0.82) | 123 (1.04) |
| **Germany** | 40564 | 274 (0.96) | 365 (1.28) | 351 (1.23) | 540 (1.89) | 587 (2.06) | 830 (2.90) | 177 (0.62) | 190 (0.66) | 204 (0.72) | 209 (0.73) |
| **Denmark** | 35220 | 219 (0.90) | 288 (1.20) | 300 (1.22) | 657 (2.70) | 508 (2.08) | 889 (3.69) | 139 (0.57) | 165 (0.68) | 146 (0.60) | 180 (0.74) |
| **Sweden** | 35330 | 288 (1.35) | 378 (1.72) | 260 (1.20) | 497 (2.22) | 518 (2.42) | 824 (3.74) | 187 (0.87) | 201 (0.91) | 181 (0.84) | 222 (1.00) |
| **Finland** | 15948 | 63 (0.85) | 101 (1.27) | 53 (0.71) | 126 (1.57) |  |  | 38 (0.51) | 49 (0.61) | 73 (0.99) | 89 (1.12) |
| **Israel** | 19472 | 45 (0.47) | 83 (0.89) | 59 (0.61) | 140 (1.49) | 100 (1.03) | 213 (2.28) | 31 (0.32) | 28 (0.30) | 47 (0.49) | 69 (0.74) |
| **Total** | **881198** | **3913 (0.87)** | **5208 (1.13)** | **3712 (0.78)** | **6540 (1.33)** | **6932 (1.56)** | **10275 (2.26)** | **2203 (0.49)** | **2677 (0.58)** | **3981 (0.88)** | **5130 (1.11)** |

ACD, all-cause death; HHF, hospitalization for heart failure; ITT, intent-to-treat; MI, myocardial infarction; oGLD, other glucose-lowering drug; SGLT-2i, Sodium-glucose cotransporter-2 inhibitor

**Figure S1. Flow chart for all countries combined**

**
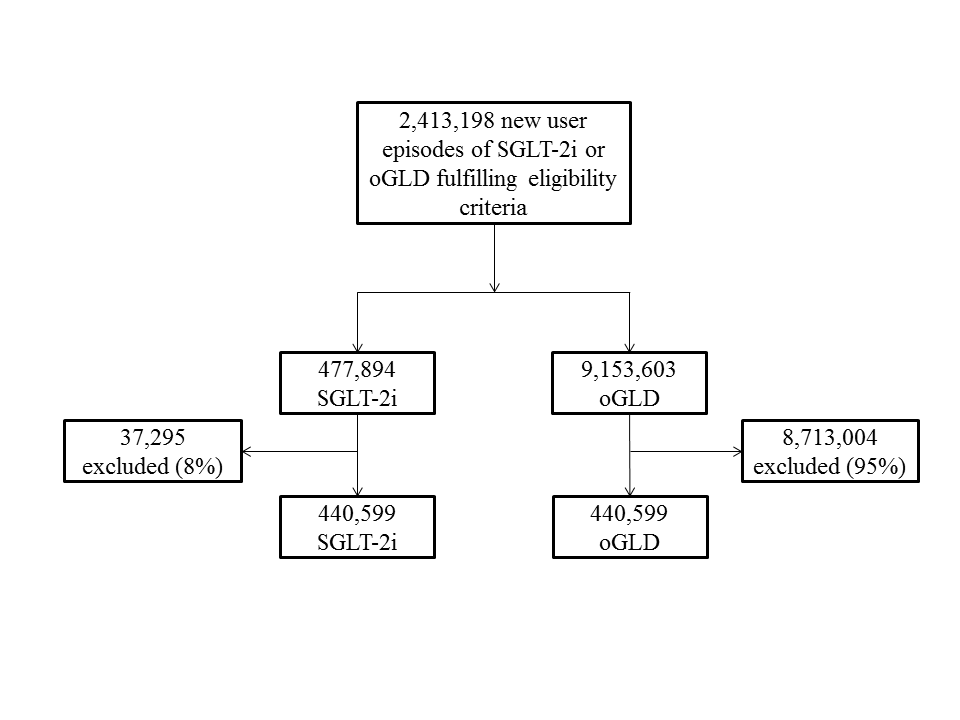
**

oGLD, other glucose-lowering drug; SGLT-2i, sodium-glucose cotransporter-2 inhibitors

**Figure S2. Pooled hazard ratios for the outcomes of (A) hospitalization for heart failure, (B) all-cause death, (C) composite of all-cause death or hospitalization for heart failure, (D) myocardial infarction, and (E) stroke (ITT, unadjusted)**

**A.**


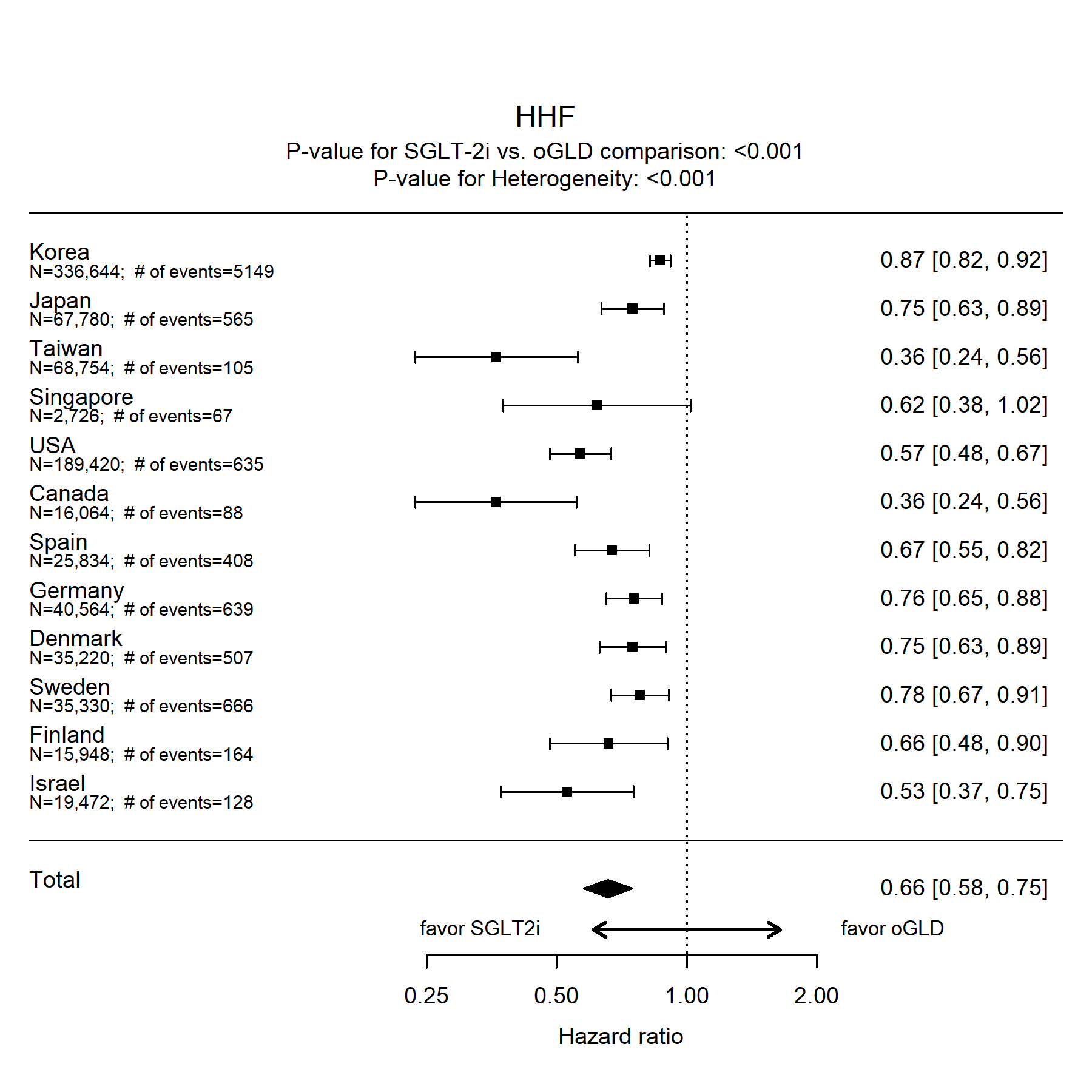


**B.**


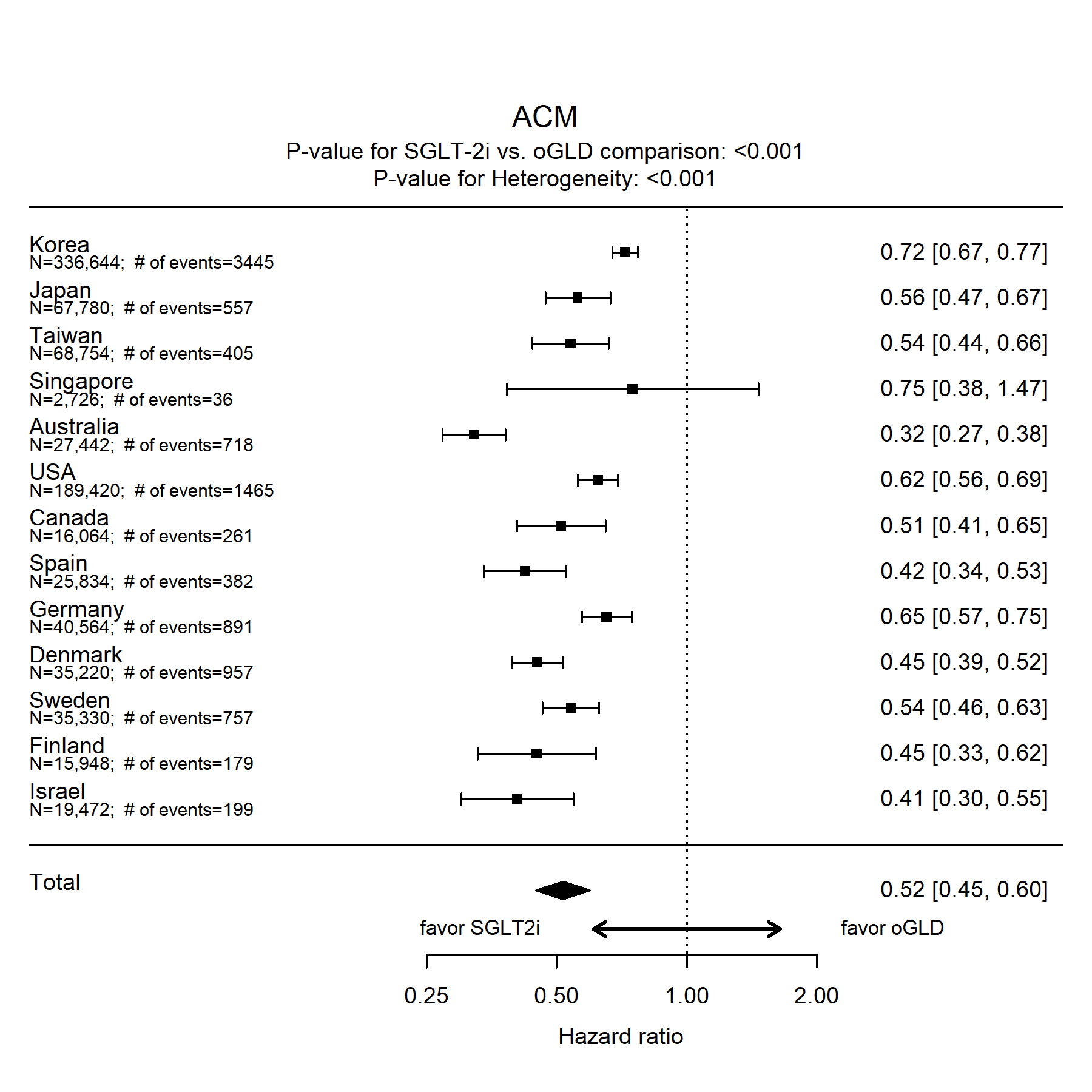


**C.**


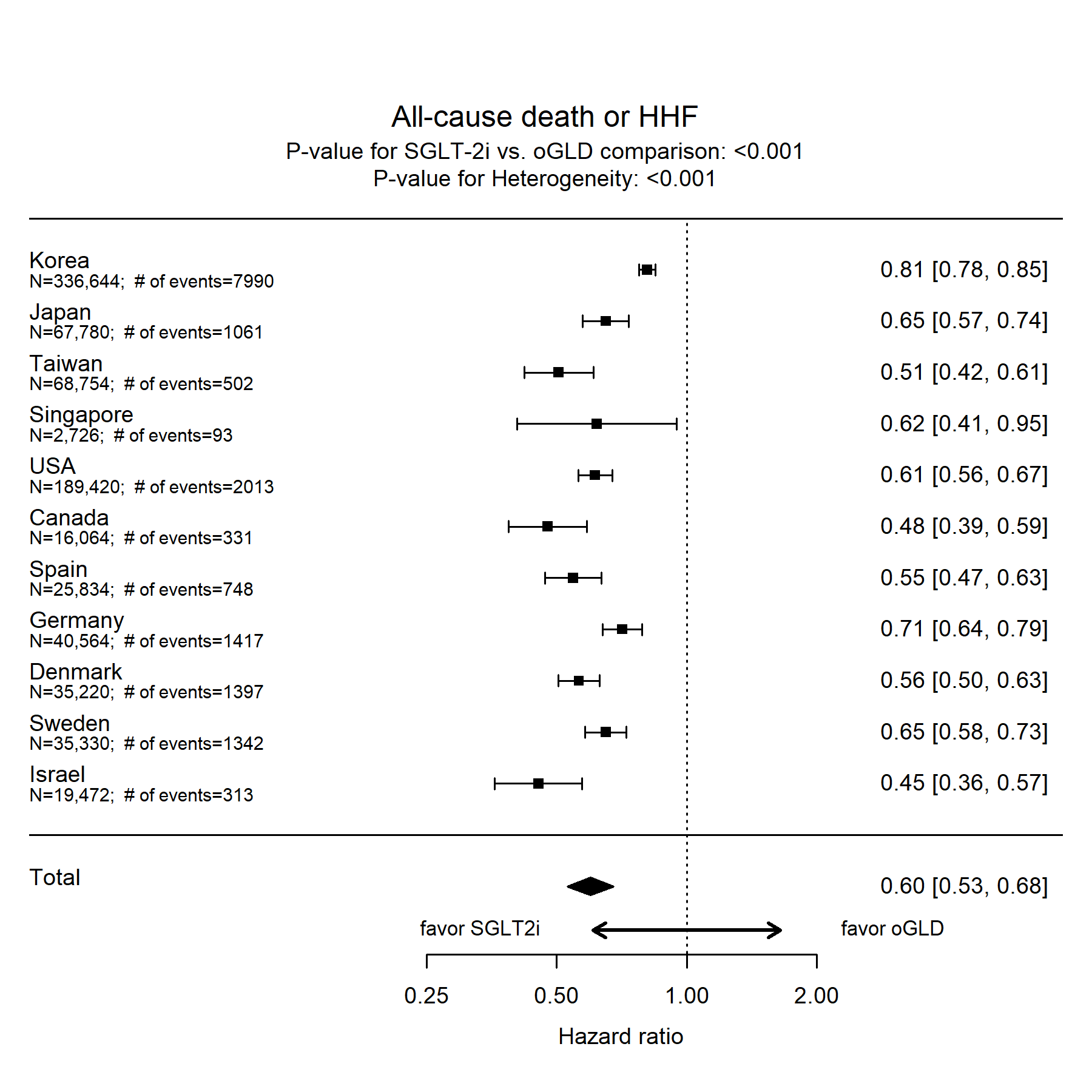


**D.**


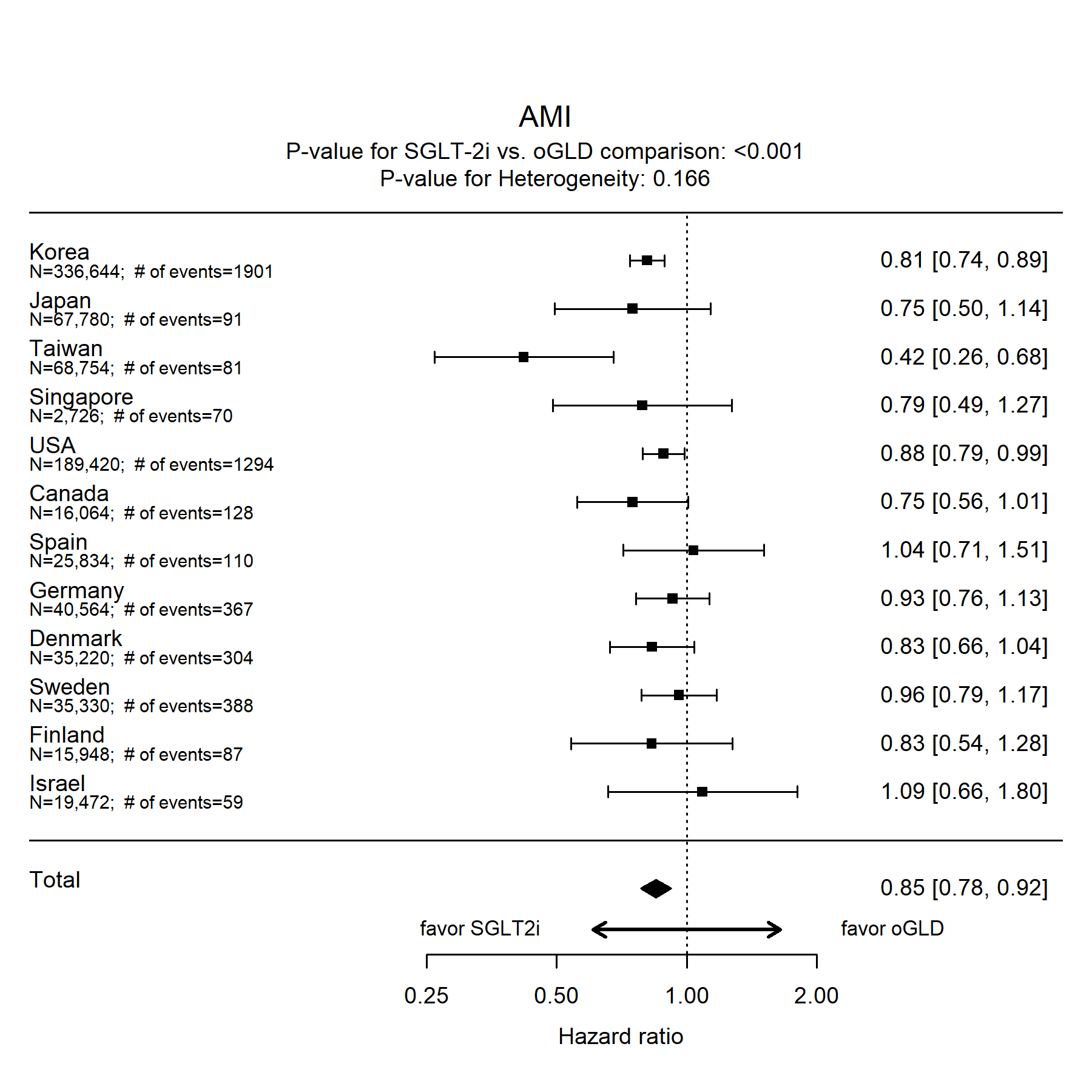


**E.**


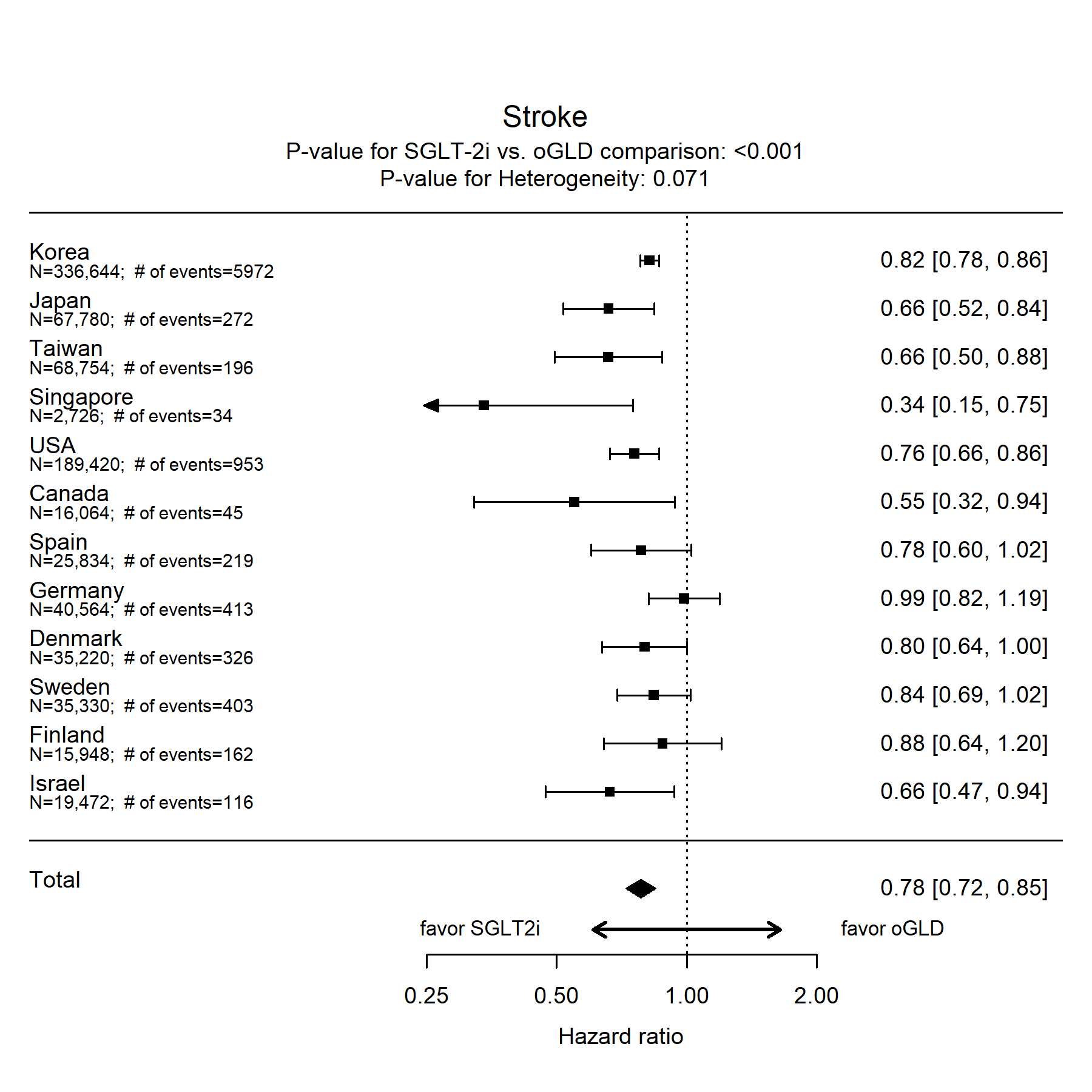


ITT, intent-to-treat; oGLD, other glucose-lowering drug; SGLT-2i, Sodium-glucose cotransporter-2 inhibitor

**Figure S3. Pooled hazard ratios for the outcomes of hospitalization for heart failure, all-cause death, composite of all-cause death or hospitalization for heart failure, myocardial infarction, and stroke (ITT, adjusted)**


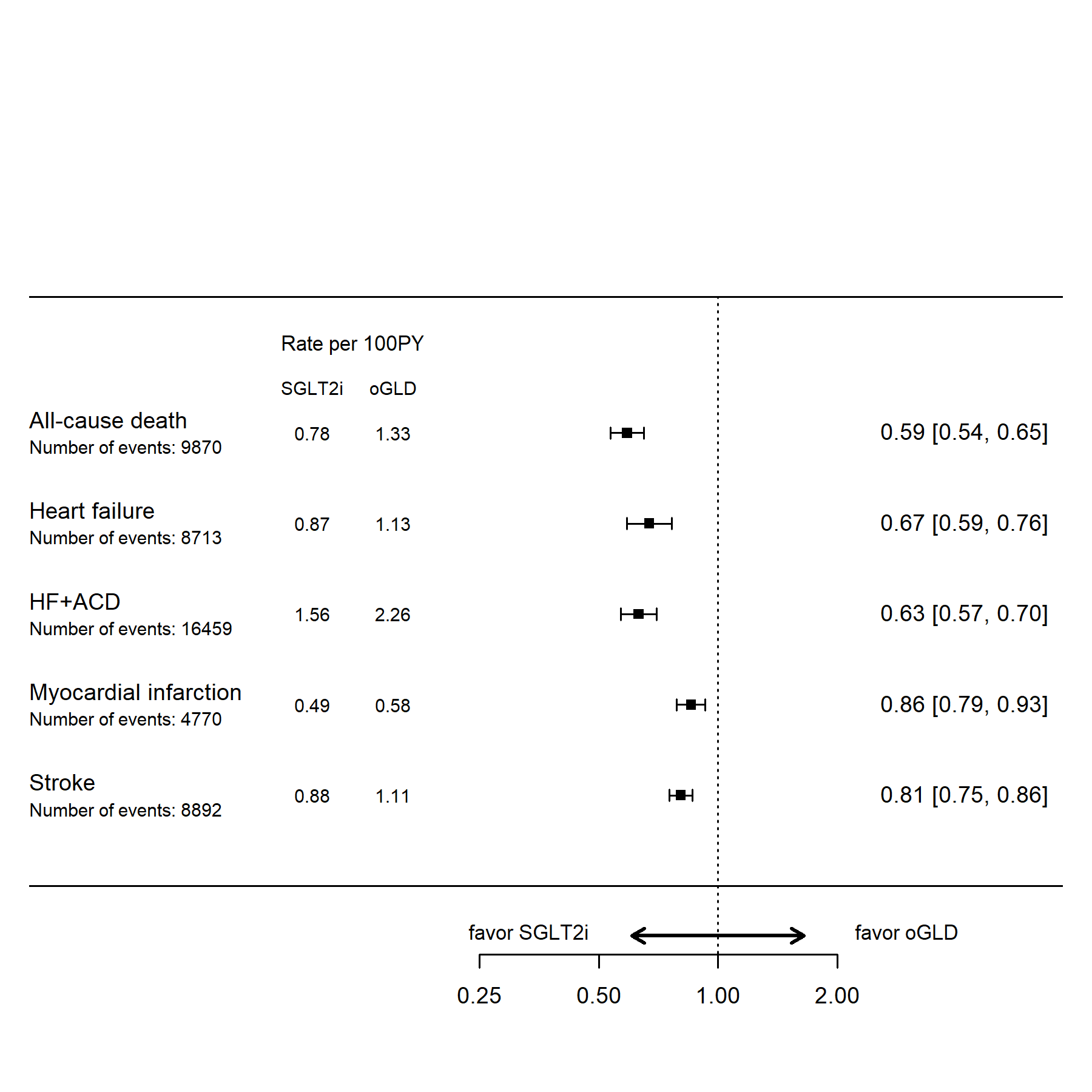


ACD, all-cause death; HF, heart failure; ITT, intent-to-treat; oGLD, other glucose-lowering drug; SGLT-2i, Sodium-glucose cotransporter-2 inhibitor

**Figure S4. Pooled hazard ratios for the outcomes of hospitalization for heart failure, all-cause death, composite of all-cause death or hospitalization for heart failure, myocardial infarction, and stroke (on treatment, unadjusted)**


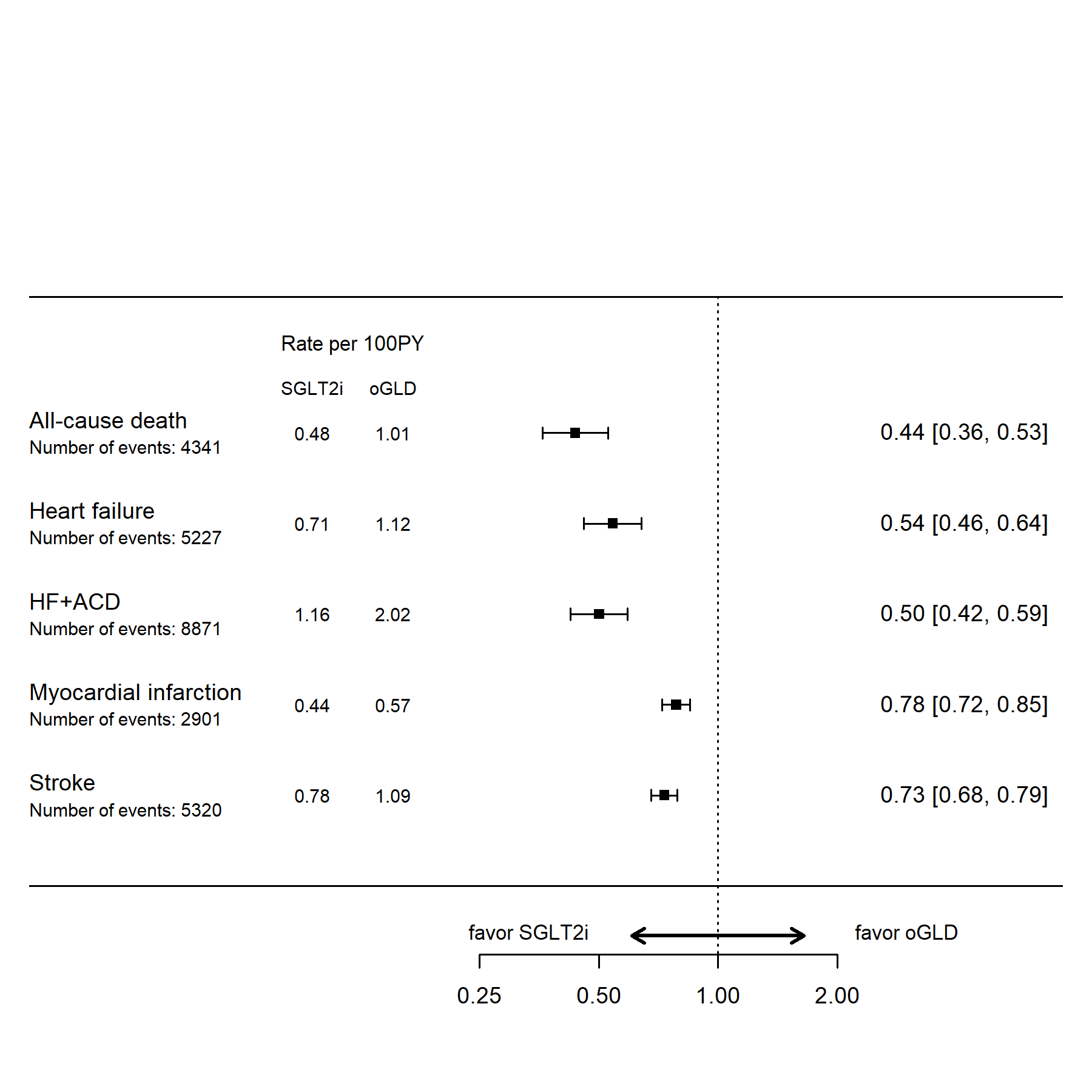


ACD, all-cause death; HF, heart failure; ITT, intent-to-treat; oGLD, other glucose-lowering drug; SGLT-2i, Sodium-glucose cotransporter-2 inhibitor

**Figure S5. Pooled hazard ratios for the outcomes of hospitalization for heart failure, all-cause death, composite of all-cause death or hospitalization for heart failure, myocardial infarction, and stroke (on treatment, adjusted)**


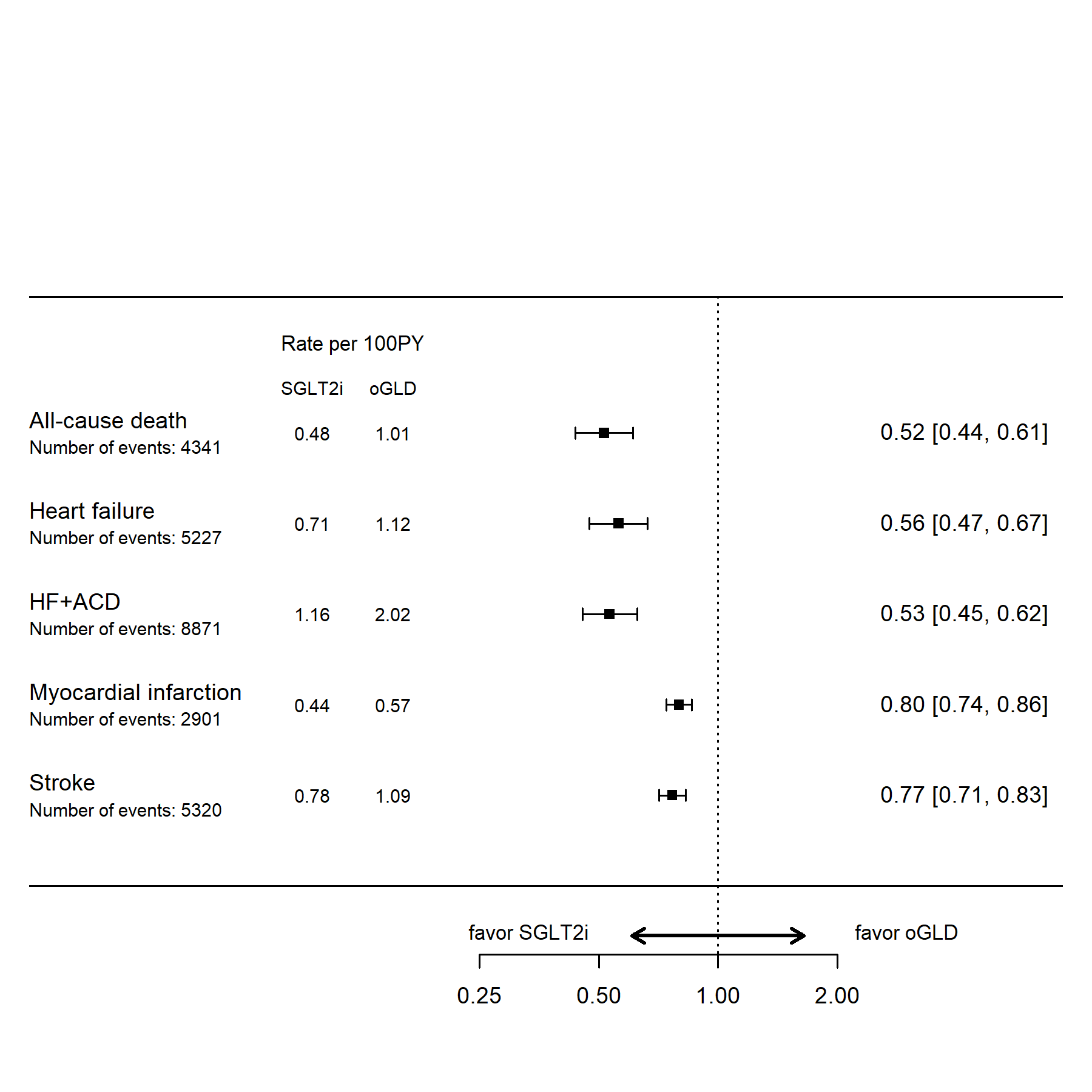


ACD, all-cause death; HF, heart failure; ITT, intent-to-treat; oGLD, other glucose-lowering drug; SGLT-2i, Sodium-glucose cotransporter-2 inhibitor
